# Supplementary material for: Receptor-interacting protein kinase 2 (RIPK2) stabilizes c-Myc and is a therapeutic target in prostate cancer metastasis
Source: Nat Commun. 2022 Feb 3;13:669. doi: 10.1038/s41467-022-28340-6 (PMC8813925; doi:10.1038/s41467-022-28340-6)
Supplement: Supplementary file 1 — Supplementary Information [file 41467_2022_28340_MOESM1_ESM.pdf]

SUPPLEMENTARY INFORMATION

Receptor-interacting Protein Kinase 2 (RIPK2) Stabilizes c-Myc and is a Therapeutic Target in Prostate Cancer Metastasis

Yiwu Yan[1, †], Bo Zhou[1,9,†], Chen Qian[1], Alex Vasquez[1], Mohini Kamra[2], Avradip Chatterjee[3], Yeon-Joo Lee[1], Xiaopu Yuan[4], Leigh Ellis[3,5,6], Dolores Di Vizio[1,3,4], Edwin M. Posadas[5,6], Natasha Kyprianou[7], Beatrice S. Knudsen[3,4,6,10], Kavita Shah[2], Ramachandran Murali[3,6], Arkadiusz Gertych[1,4], Sungyong You[1,3,6], Michael R. Freeman[1,3,6,8], Wei Yang[1,3,6,8,*]

[1]Department of Surgery, Cedars-Sinai Medical Center, Los Angeles, California, United States
[2]Department of Chemistry and Purdue University Center for Cancer Research, Purdue University, West Lafayette, Indiana, United States
[3]Department of Biomedical Sciences, Cedars-Sinai Medical Center, Los Angeles, California, United States
[4]Department of Pathology and Laboratory Medicine, Cedars-Sinai Medical Center, Los Angeles, California, United States
[5]Department of Medicine, Cedars-Sinai Medical Center, Los Angeles, California, United States
[6]Samuel Oschin Comprehensive Cancer Institute, Cedars-Sinai Medical Center, Los Angeles, California, United States
[7]Department of Urology, Icahn School of Medicine at Mount Sinai, New York, New York, United States
[8]Department of Medicine, University of California Los Angeles, Los Angeles, California, United States
[9]Present address: InterVenn Biosciences, South San Francisco, California, United States
[10]Present address: Department of Pathology, University of Utah, Salt Lake City, Utah, United States
[†]These authors contributed equally
[*]Corresponding author. Tel: +1 (310)423-7142; Email: wei.yang@cshs.org

Inventory of Supplementary Information

Supplementary Figures 1-41

1. Supplementary Figure 1. Related to Figure 1. Expression levels of RIPK2.
2. Supplementary Figure 2. Related to Figure 1. *RIPK2* is frequently amplified or overexpressed in multiple cancer types.
3. Supplementary Figure 3. Related to Figure 1. *RIPK2* overexpression is associated with significantly shorter overall survival in nine cancer types in the TCGA PanCancer Atlas.
4. Supplementary Figure 4. Related to Figure 2. *RIPK2* knockout has a negligible effect on PC cell proliferation and migration and inhibits 2D and 3D invasion *in vitro*.
5. Supplementary Figure 5. Representative flow cytometry plots showing the gating strategy.
6. Supplementary Figure 6. Related to Figure 2. *RIPK2* knockout suppresses PC metastasis *in vivo*.
7. Supplementary Figure 7. Related to Figure 2. *RIPK2* knockout modestly reduces tumor size and weight.
8. Supplementary Figure 8. Related to Figure 3. RIPK2 knockout does not inhibit the canonical RIPK2-downstream NF-κB pathway.
9. Supplementary Figure 9. Related to Figure 3. Schematic of label-free proteomics to identify proteins downstream of RIPK2.
10. Supplementary Figure 10. Related to Figure 3. Quality assessment of the label-free proteomics data.
11. Supplementary Figure 11. Related to Figure 3. Identification of differentially expressed proteins (DEPs) by *RIPK2*-KO in PC cells.
12. Supplementary Figure 12. Related to Figure 3. Bar graphs of the top 10 significantly enriched gene ontology (GO) terms identified by ToppFun analysis.
13. Supplementary Figure 13. Related to Figure 3. Line plots of the mean Z scores of the protein-set downregulated or upregulated by *RIPK2*-KO in PC tissue specimens.
14. Supplementary Figure 14. Related to Figure 3. MYC gene signatures (V1 and V2) are the most strongly downregulated hallmark signatures by *RIPK2*-KO.
15. Supplementary Figure 15. Related to Figure 3. *RIPK2*-induced activity scores and *RIPK2* mRNA abundance strongly correlate with *MYC* activity scores in PC tissue specimens.
16. Supplementary Figure 16. Related to Figure 3. Among all MSigDB hallmark gene sets, the *MYC* target gene sets (V1 and V2) are most strongly associated with *RIPK2* mRNA and activity levels in PC tissue specimens.
17. Supplementary Figure 17. Related to Figure 3. *RIPK2*-induced activity scores are strongly associated with *MYC* activity scores across 32 different cancer types.
18. Supplementary Figure 18. Related to Figure 4. The co-amplification/gain of *RIPK2* and *MYC* genes is a frequent event in multiple cancer types and synergistically contributes to c-Myc protein abundance.
19. Supplementary Figure 19. Related to Figure 4. The regulation of c-Myc by RIPK2 is mainly not at the transcriptional level.
20. Supplementary Figure 20. Related to Figure 4. Genetic knockout of *RIPK2* destabilizes the c-Myc protein by promoting its proteasomal degradation.
21. Supplementary Figure 21. Related to Figure 4. The genetic alteration of *RIPK2* is more frequent than that of any known direct c-Myc-S62 kinases in PC tissue specimens.
22. Supplementary Figure 22. Related to Figure 4. RIPK2 does not bind to c-Myc under physiological condition.
23. Supplementary Figure 23. Related to Figure 4. NLS and NES specifically target RIPK2 into the nuclei and the cytoplasm, respectively.
24. Supplementary Figure 24. Related to Figure 5. Illustration of RIPK2 complexes and predicted immunoprecipitation products.
25. Supplementary Figure 25. Related to Figure 5. Quality assessment of the immunoprecipitation samples.
26. Supplementary Figure 26. Related to Figure 5. Identification of significantly enriched proteins in eight IP-MS comparisons.
27. Supplementary Figure 27. Related to Figure 5. Identification of candidate proteins associated with the kinase domain of cytoplasmic RIPK2.
28. Supplementary Figure 28. Related to Figure 5. Phosphoproteomic identification of candidate kinases whose activities are regulated by RIPK2.
29. Supplementary Figure 29. Related to Figure 6. RIPK2 binds to and activates MKK7, a major mediator of RIPK2 regulation of c-Myc.
30. Supplementary Figure 30. Related to Figure 6. DNA-PKcs encoded by *PRKDC* is a RIPK2-interacting proteins.
31. Supplementary Figure 31. Related to Figure 6. MKK7 mediates RIPK2-induced c-Myc and JNK phosphorylation.

32. Supplementary Figure 32. Related to Figure 7. JNK only mediates a subset of RIPK2/MKK7 phosphorylation of c-Myc-S62.
33. Supplementary Figure 33. Related to Figure 7. MKK7 associates with c-Myc in the nuclei.
34. Supplementary Figure 34. Related to Figure 7. Kinase-dead MKK7-K149A is much less efficient than constitutively active MKK7-3E (S271E, T275E, S277E) in phosphorylating c-Myc-S62 *in vitro*.
35. Supplementary Figure 35. Related to Figure 8. Pharmacological inhibition of RIPK2 reduces p-JNK, p-c-Myc-S62, and c-Myc levels.
36. Supplementary Figure 36. Related to Figure 8. Pharmacological inhibition of RIPK2 suppresses PC cell invasion and colony formation.
37. Supplementary Figure 37. Related to Figure 8. GSK583 or ponatinib only has modest effects on PC cell viability when used at $\leq$10 or $\leq$1 μM, respectively.
38. Supplementary Figure 38. Related to Figure 8. GSK583 inhibits PC metastasis *in vivo*.
39. Supplementary Figure 39. Related to Figure 8. GSK583 inhibits PC liver metastasis *in vivo*.
40. Supplementary Figure 40. Related to Figure 8. Ponatinib is more potent than commercially available preclinical RIPK2 inhibitors in inactivating MKK7/c-Myc signaling.
41. Supplementary Figure 41. Related to Figure 8. Ponatinib inhibits PC metastasis in a dose-dependent fashion.

Supplementary Tables 1-3

1. Supplementary Table 1. List of commercially available small-molecule RIPK2 inhibitors.
2. Supplementary Table 2. List of primer sequences for molecular cloning.
3. Supplementary Table 3. List of sgRNA sequences for CRISPR/Cas9 knockout.

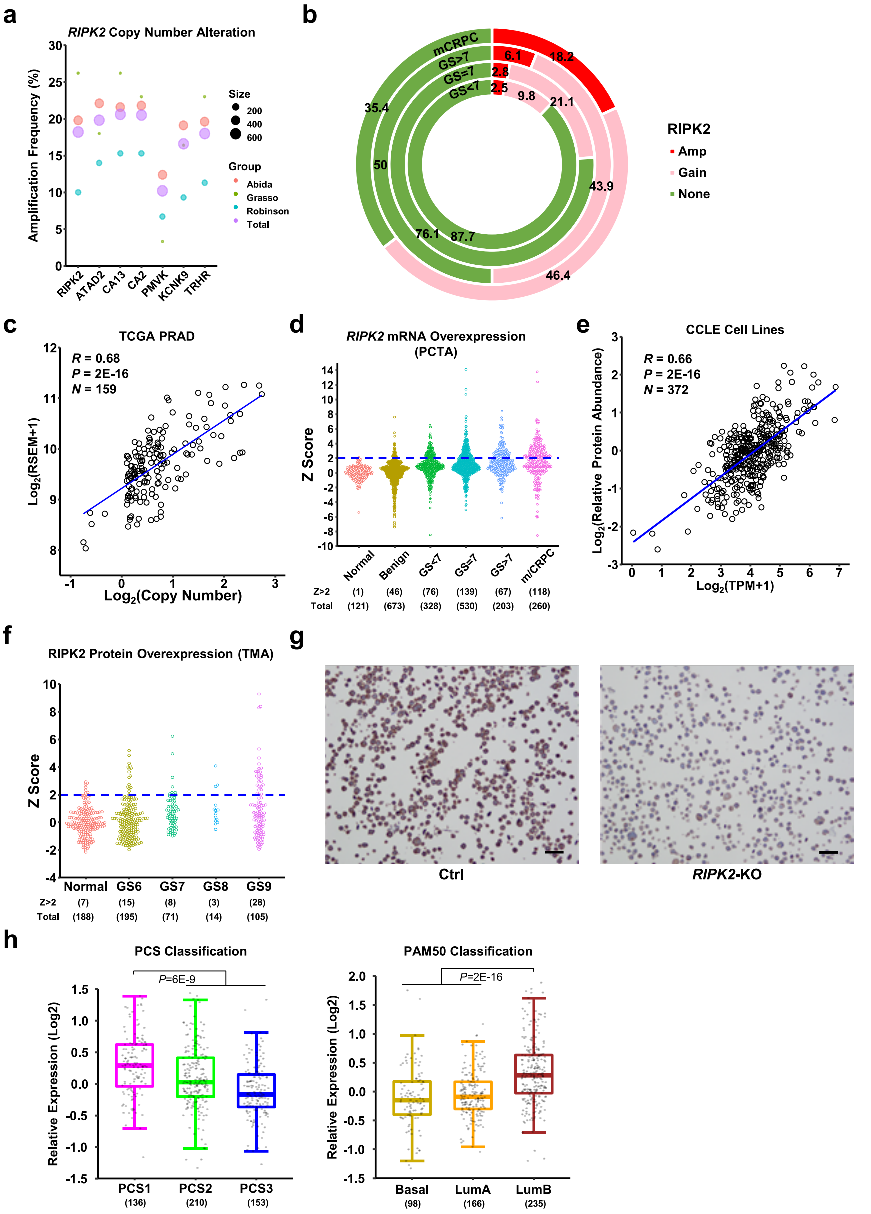

Supplementary Figure 1. Expression levels of RIPK2. a Scatter plot of the copy number amplification frequencies of the indicated genes in three independent mCRPC cohorts and on average. **b** Donut chart of the frequencies (%) of copy number alterations of *RIPK2* in four different groups of PC samples. "None" indicates without *RIPK2* amplification or gain. **c** Correlation of *RIPK2* copy numbers and mRNA abundance in the TCGA PC cohort. **d** Swarm plot of *RIPK2* mRNA abundance, which was *Z*-transformed relative to tumor-adjacent normal tissue, in the indicated groups of the PCTA tissue specimens. **e** Correlation of *RIPK2* mRNA abundance and protein abundance in cancer cells contained in the Cancer Cell Line Encyclopedia (CCLE). **f** Swarm plot of RIPK2 protein abundance, which was *Z*-transformed relative to tumor-adjacent normal tissue, in the indicated groups of tissue specimens contained in a PC tissue microarray. **g** Representative images of immunohistochemistry (IHC) staining of RIPK2 in control (left) or *RIPK2*-KO (right) HEK293T cells. Scale bars, 50 µm. **h** Box plots of *RIPK2* mRNA levels in the PCS (left) and PAM50 (right) subtypes of PC tumors in the TCGA Firehose Legacy Cohort (n = 499). Box plots indicate median (middle line), 25th and 75th percentile (box), and 5th and 95th percentile (whiskers). Nominal *p*-values were determined by two-sided rank-sum test. For correlation analyses (panels c and e), correlation coefficient (*R*) was computed by Pearson's method, and significance (*P*) was computed based on *t*-distribution. Numbers in parentheses indicate sample sizes (panels d, f, and h).

a

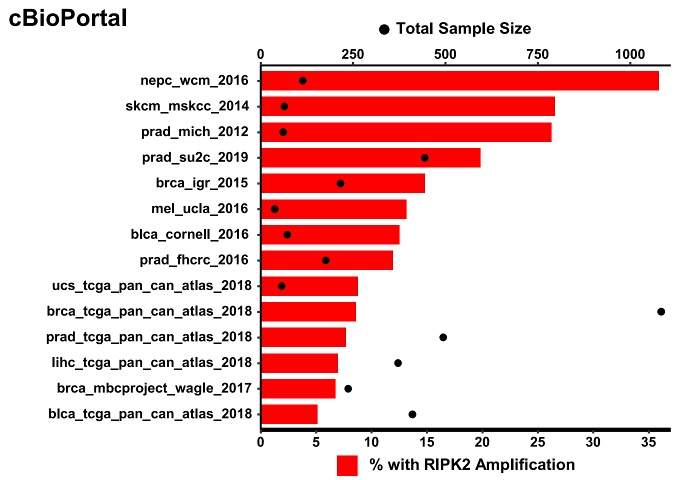

b

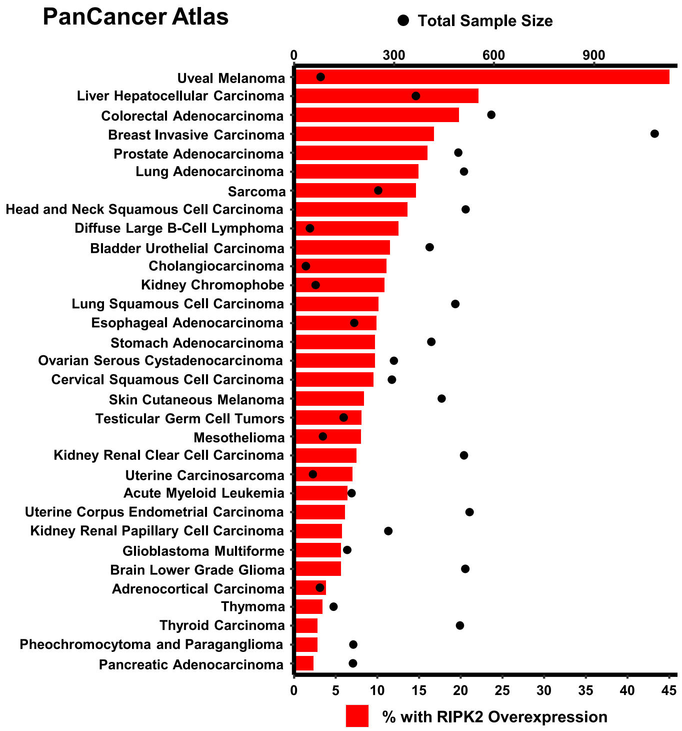

Supplementary Figure 2. *RIPK2* is frequently amplified or overexpressed in multiple cancer types. a Bar plot of the percentages of tumors with *RIPK2* gene amplification in different cancer genomics studies (retrieved from the cBioPortal). The "total sample size", shown in black dots, represents the number of tissue specimens with copy number data. Bar lengths correspond to the percentages of tumors with *RIPK2* amplification. **b** Bar plot of the percentages of tumors with *RIPK2* mRNA overexpression (Z > 2, relative to diploid samples) in the 32 TCGA PanCancer Atlas studies. The "total sample size", shown in black dots, represents the number of tissue specimens with mRNA expression data. Bar lengths correspond to the percentages of tumors with *RIPK2* mRNA overexpression (Z > 2).

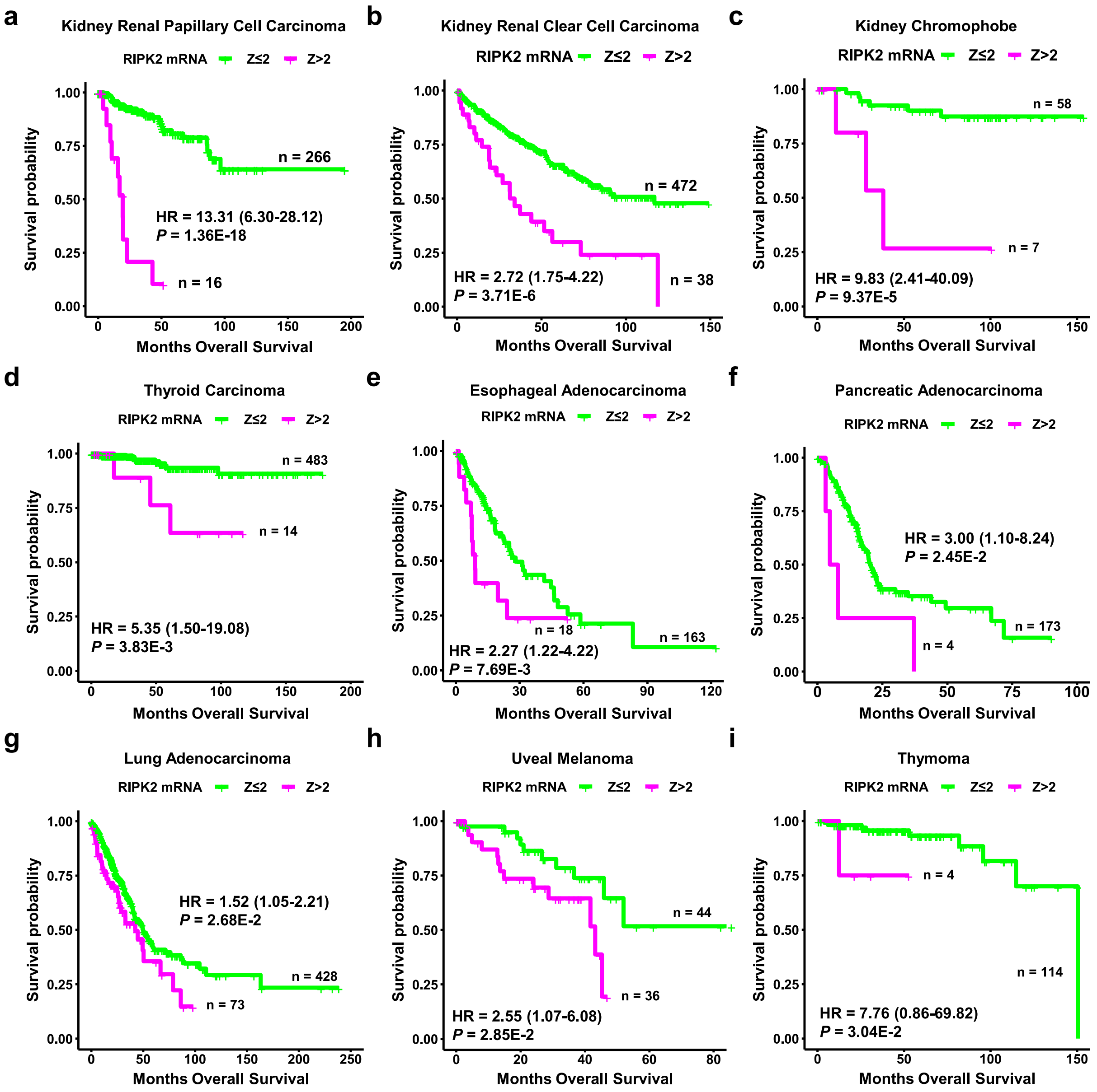

Supplementary Figure 3. *RIPK2* overexpression is associated with significantly shorter overall survival in nine cancer types in the TCGA PanCancer Atlas. a Kaplan-Meier overall survival analysis of kidney papillary cell carcinoma (n = 282). **b** Kaplan-Meier overall survival analysis of kidney clear cell carcinoma (n = 510). **c** Kaplan-Meier overall survival analysis of kidney chromophobe (n = 65). **d** Kaplan-Meier overall survival analysis of thyroid carcinoma (n = 497). **e** Kaplan-Meier overall survival analysis of esophageal adenocarcinoma (n = 181). **f** Kaplan-Meier overall survival analysis of pancreatic adenocarcinoma (n = 177). **g** Kaplan-Meier overall survival analysis of lung adenocarcinoma (n = 501). **h** Kaplan-Meier overall survival analysis of uveal melanoma (n = 80). **i** Kaplan-Meier overall survival analysis of thymoma (n = 118). Patients were stratified based on whether *RIPK2* is significantly ($Z > 2$, normalized against diploid samples) overexpressed. Nominal *p*-values were determined by two-sided log-rank test. HR, hazard ratio (95% confidence interval).

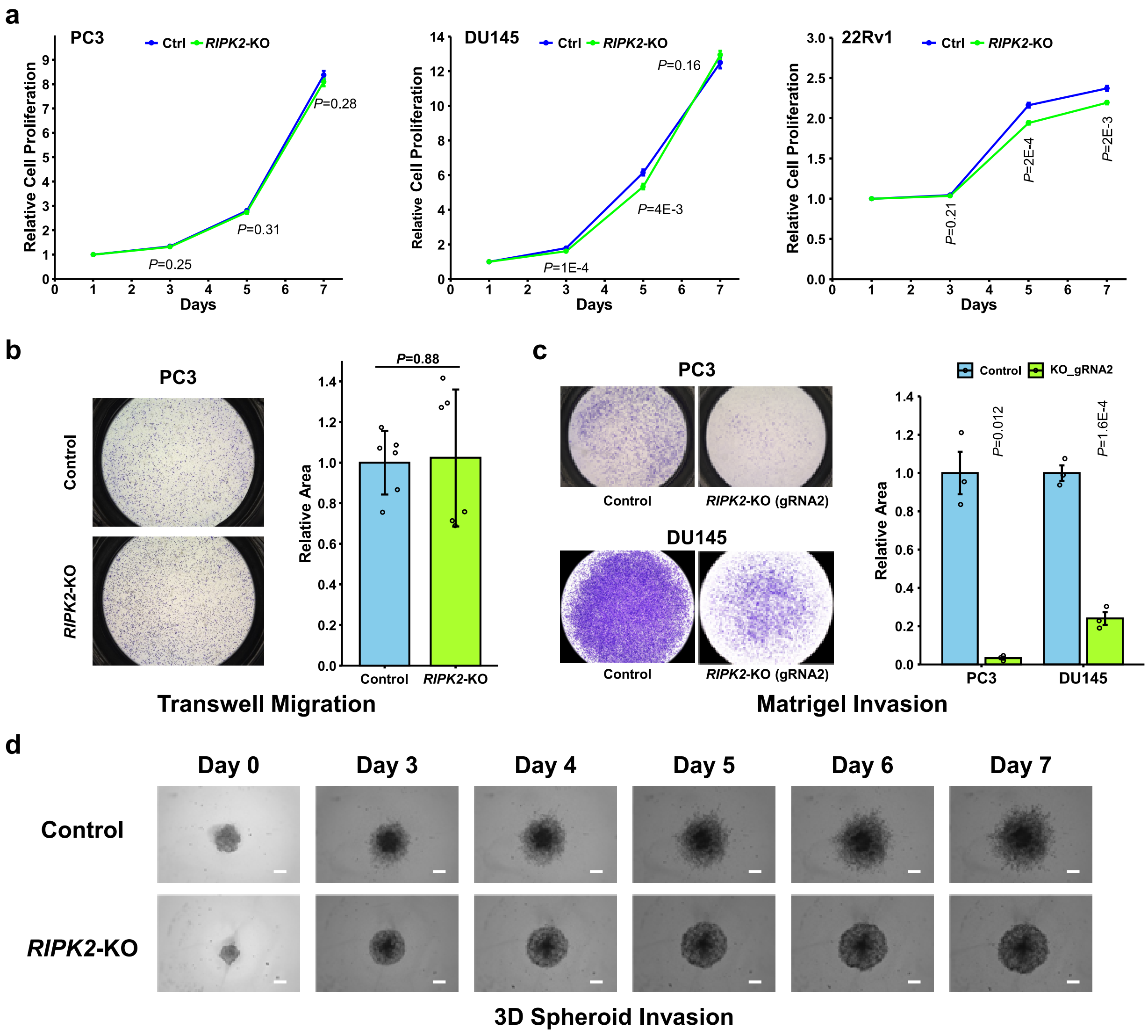

Supplementary Figure 4. *RIPK2* knockout has a negligible effect on PC cell proliferation and migration but inhibits 2D and 3D invasion *in vitro*. a Growth curves of control and *RIPK2*-KO PC3 (left), DU145 (middle), and 22Rv1 (right) cells (n = 10 biologically independent samples per group). **b** Representative images (left) and quantification (right) of transwell migration assay of control or *RIPK2*-KO PC3 cells (n = 6 biologically independent samples per group). **c** Representative images (left) and quantification (right) of Matrigel invasion assay of control PC cells and cells with *RIPK2* KO by CRISPR/Cas9 using gRNA2 (n = 3 biologically independent samples per group). **d** Representative images of 3D spheroid invasion assay of control or *RIPK2*-KO PC3 cells (n = 6 biologically independent samples per group). Scale bars, 300 µm. Nominal *p*-values were determined by two-sided Mann-Whitney *U* test (panel a) or unpaired two-tailed Student's *t*-test (panels b and c). Data are Mean ± standard deviation (SD).

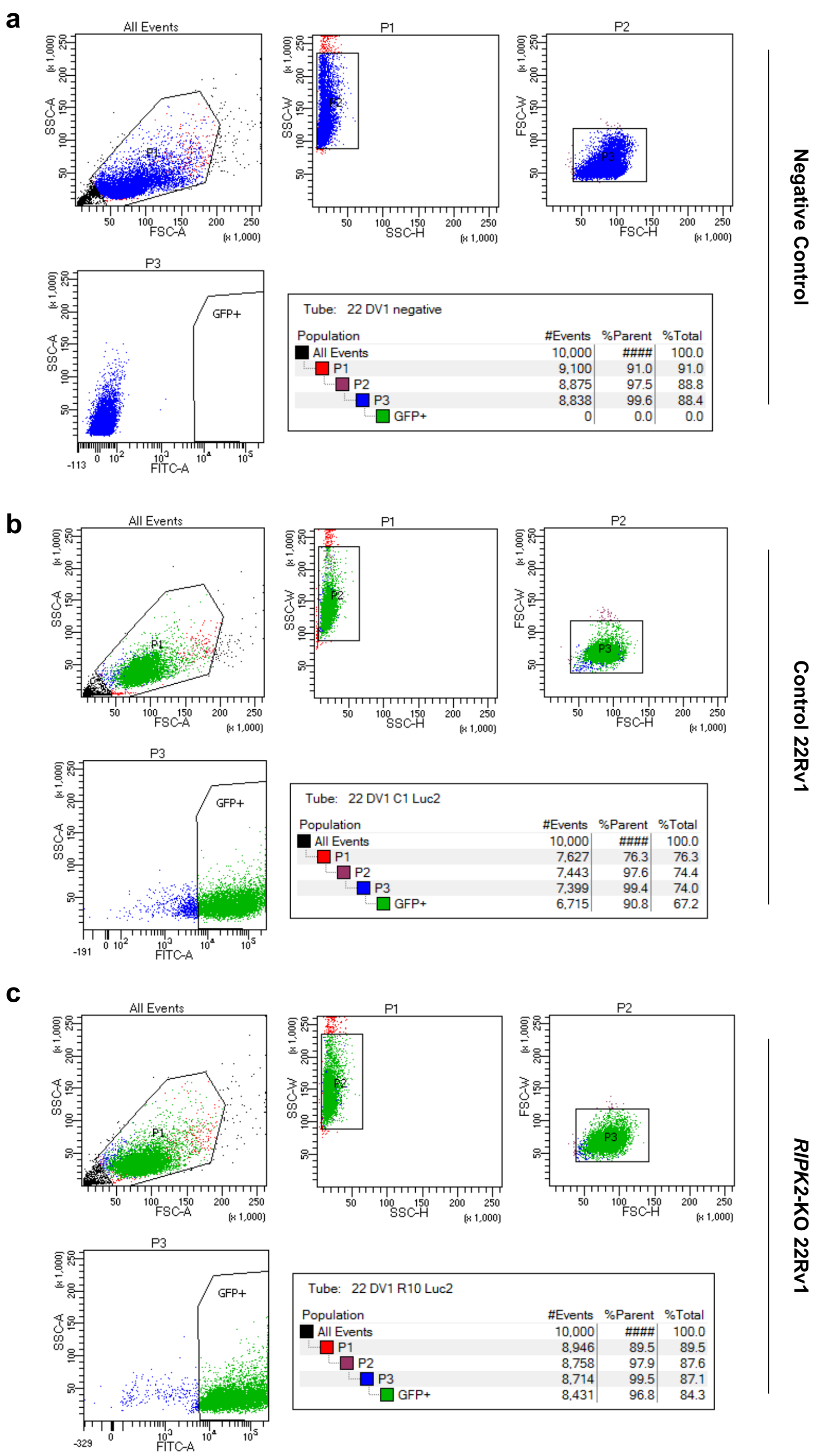

Supplementary Figure 5. Representative flow cytometry plots showing the gating strategy. **a** Flow cytometry plot of parental 22Rv1 cells. **b** Flow cytometry plot of luciferase-expressing control 22Rv1 cells. **c** Flow cytometry plot of luciferase-expressing *RIPK2*-KO 22Rv1 cells.

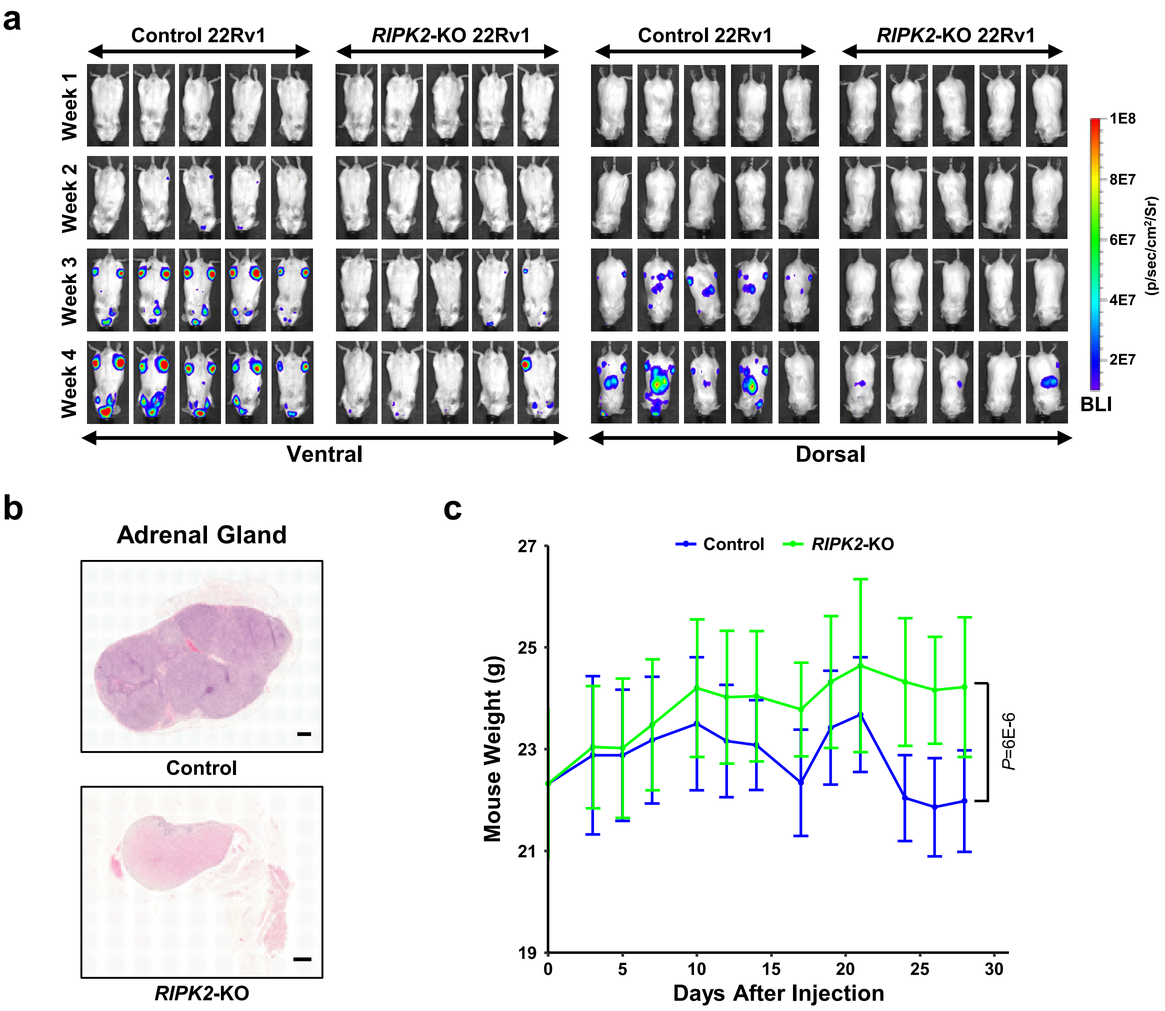

Supplementary Figure 6. *RIPK2* knockout suppresses PC metastasis *in vivo*. a Bioluminescence images of mice from the ventral (upper) and dorsal (lower) sides at different weeks after the intracardiac injection. **b** Representative images of H&E staining of adrenal glands from mice bearing control or *RIPK2*-KO 22Rv1 cells. Scale bars, 300 µm. **c** Changes in mouse weights following the intracardiac injection. Nominal *p*-value was determined by two-way ANOVA test (panel c). Data are Mean ± SEM (n = 5 biologically independent mice per group) (panel c).

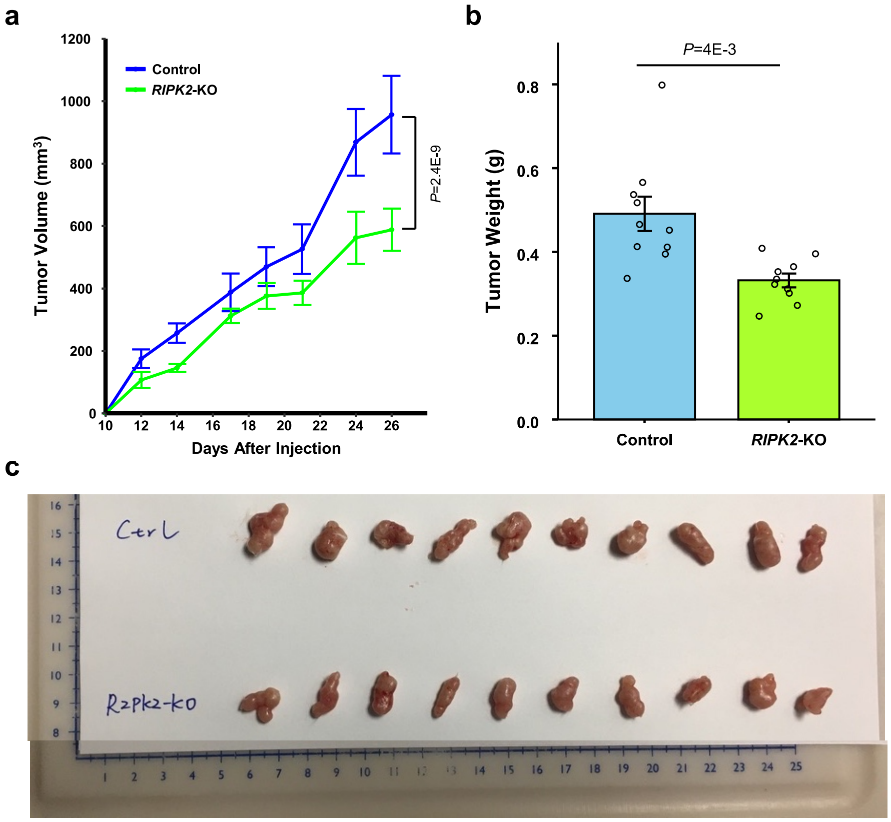

Supplementary Figure 7. *RIPK2* knockout modestly reduces tumor size and weight. a Effect of *RIPK2*-KO on 22Rv1 xenograft tumor growth following subcutaneous injection of control or *RIPK2*-KO 22Rv1 cells into male SCID/beige mice (n = 10 biologically independent tumors per group). **b** Bar plot of the weights of 22Rv1 control or *RIPK2*-KO tumors on day 26 after subcutaneous injection (n = 10 biologically independent tumors per group). **c** Images of xenograft tumors derived from control (upper) or *RIPK2*-KO (lower) 22Rv1 cells. Tumors were harvested 26 days after subcutaneous injection. Nominal *p*-values were determined by two-way ANOVA (panel a) or unpaired two-tailed Student's *t*-test (panel b). Data are Mean ± SEM (panels a and b).

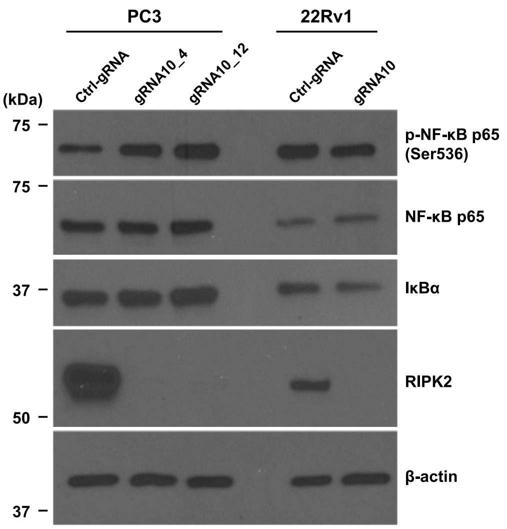

Supplementary Figure 8. ***RIPK2*** **knockout does not inhibit the canonical RIPK2-downstream NF-κB pathway.** Representative immunoblot of p-p65 (a surrogate for NF-κB p65 activity), NF-κB p65, and IκBα (an endogenous inhibitor of NF-κB) in total lysates of control or *RIPK2*-KO PC3 and 22Rv1 cells. β-actin was used as a loading control.

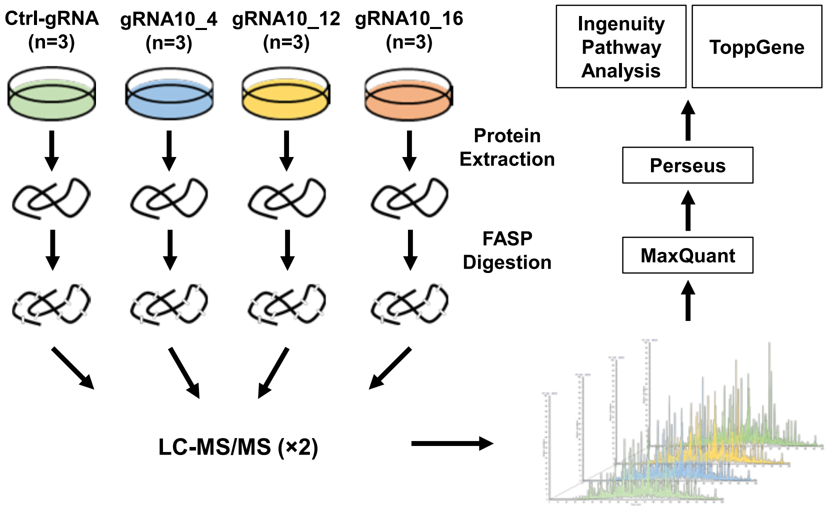

Supplementary Figure 9. Schematic of label-free proteomics to identify proteins downstream of RIPK2. gRNA10_4, gRNA10_12, and gRNA10_16 represent *RIPK2*-KO (by gRNA#10) PC3 single-cell clones 4, 12, and 16, respectively. FASP, filter-aided sample preparation; LC-MS/MS, liquid chromatography-tandem mass spectrometry.

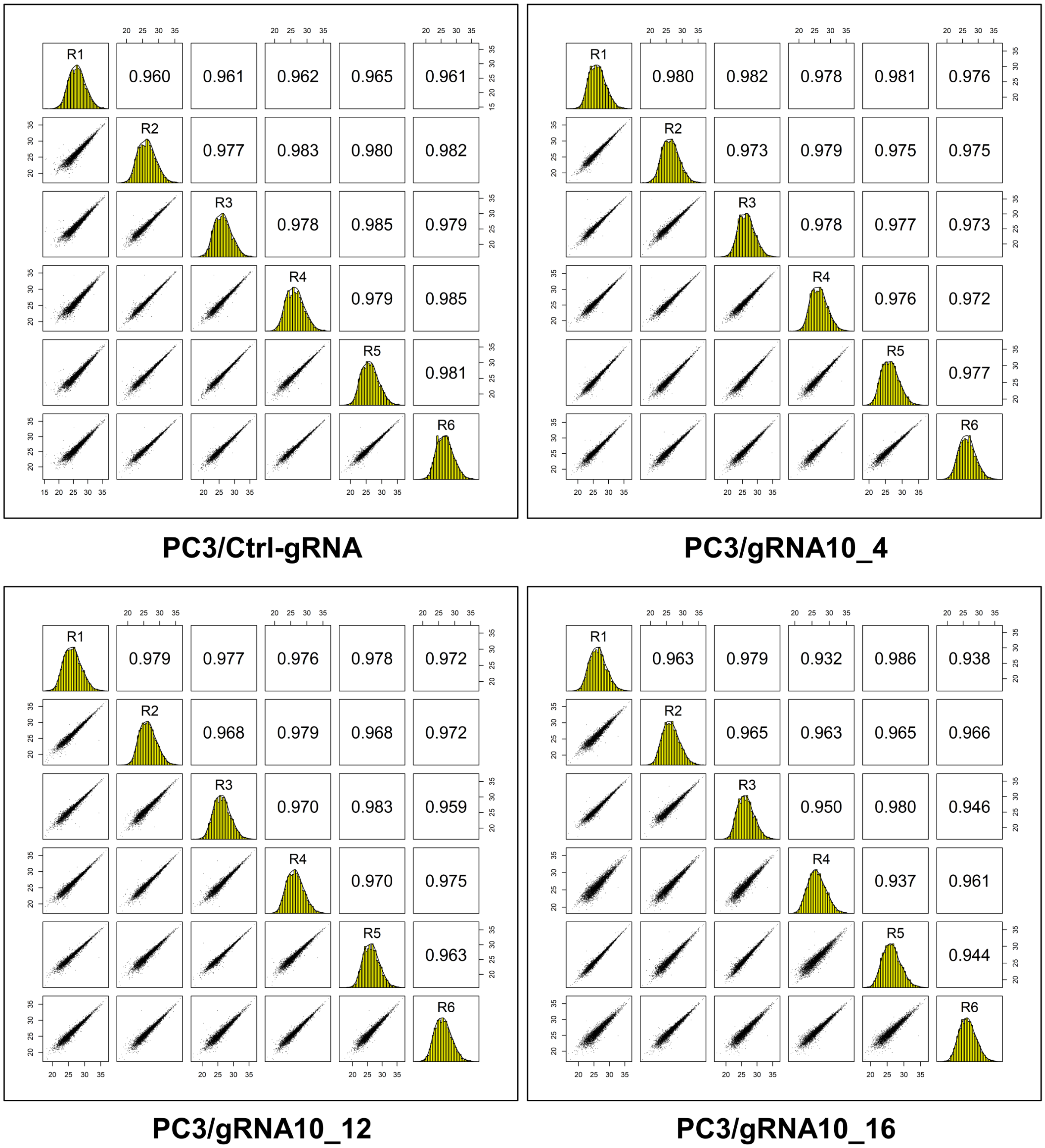

Supplementary Figure 10. Quality assessment of the label-free proteomics data. PC3/Ctrl-gRNA (upper left): PC3 cells stably transfected with a non-targeting control gRNA. PC3/gRNA10_4 (upper right): PC3 cells stably transfected with *RIPK2*-targeting gRNA#10, single clone #4. PC3/gRNA10_12 (lower left): PC3/gRNA10, single clone #12. PC3/gRNA10_16 (lower right): PC3/gRNA10, single clone #16. Numbers in boxes: Pearson correlation coefficients of log2-transformed label-free quantification (LFQ) intensities of protein groups between replicates. Histograms: distribution of log_2(LFQ) values of protein groups in each replicate. R1-R6: replicates #1-#6. Scatter plots: distribution of log_2(LFQ) values of protein groups in two different replicates.

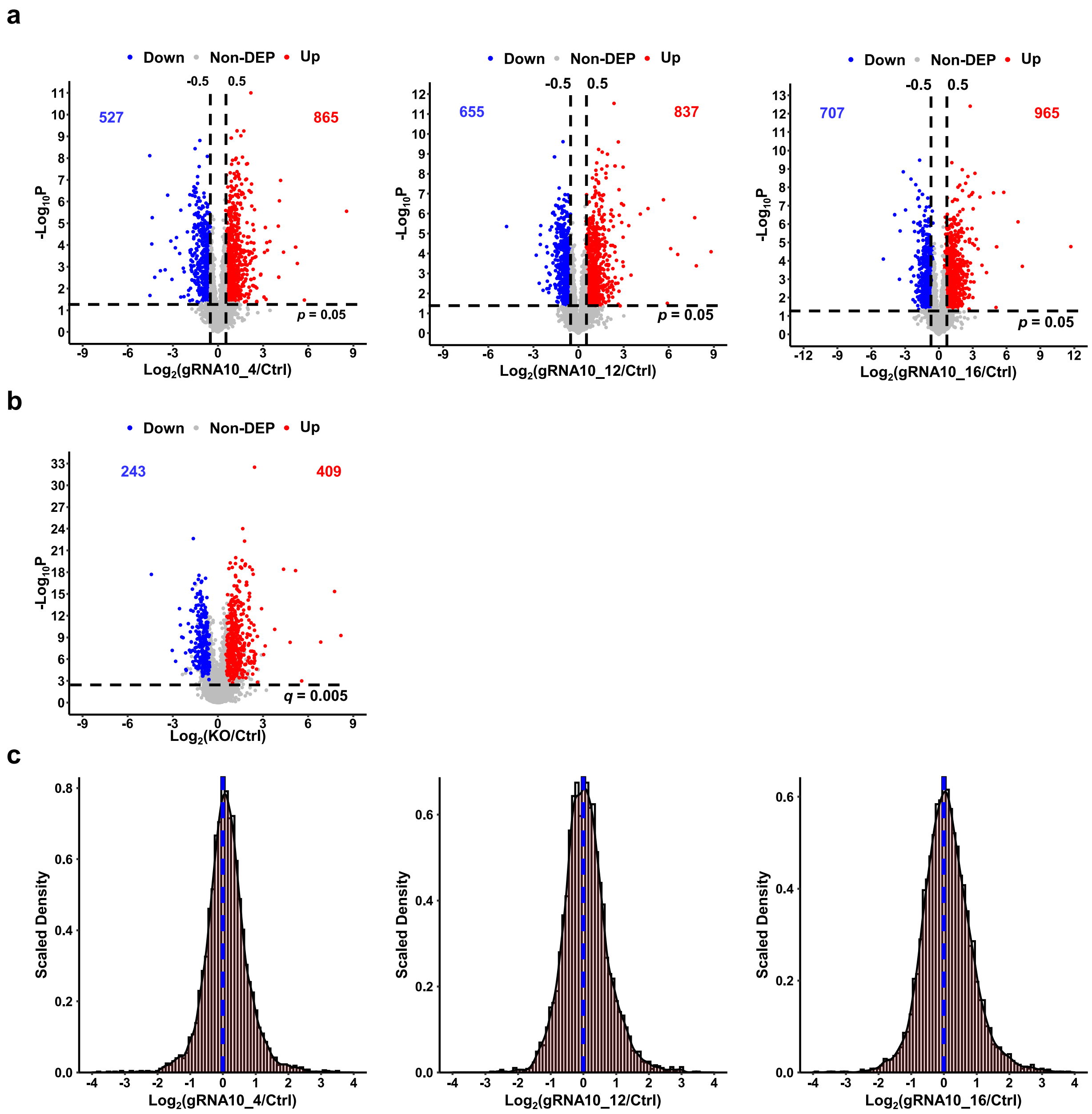

Supplementary Figure 11. Identification of differentially expressed proteins (DEPs) by *RIPK2*-KO in PC3 cells. **a** Volcano plots of all protein groups quantified by label-free proteomics comparison of control PC3 cells and *RIPK2*-KO PC3 single-cell clones #4 (left), #12 (middle), or #16 (right). **b** Volcano plot comprising the 652 DEPs consistently identified from all the three *RIPK2*-KO single-cell clones, compared with control PC3 cells. The x-axis shows the average $\log_2$(KO/Ctrl) values across the three *RIPK2*-KO clones. The y-axis shows the negative $\log_{10}$-transformed combined nominal *p*-values. The *q*-value cutoff of 0.005 corresponds to combined nominal *p*-value of 0.00187. **c** Density plots of $\log_2$-transformed protein ratios between *RIPK2*-KO PC3 single-cell clones and control PC3 cells. Nominal *p*-values were determined by two-tailed Student's *t*-test (panels a and b).

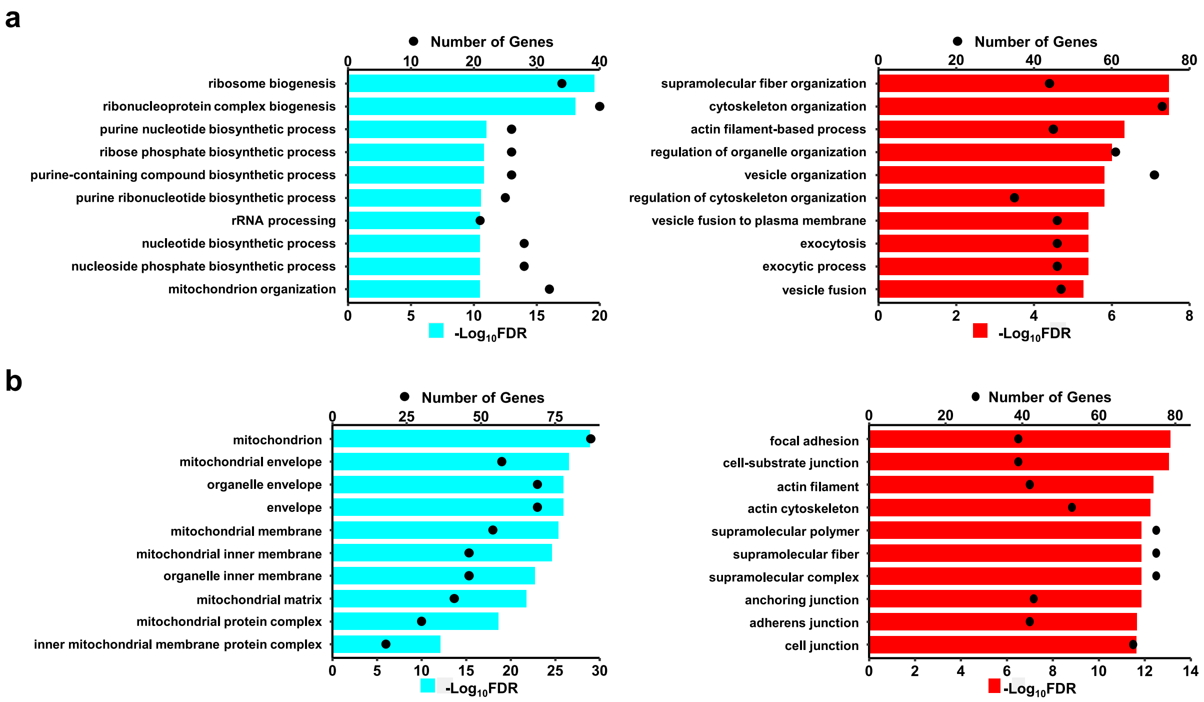

Supplementary Figure 12. Bar graphs of the top 10 significantly enriched gene ontology (GO) terms identified by ToppFun analysis. a Bar plots of the top 10 significantly enriched biological processes (GO_BP). **b** Bar plots of the top 10 significantly enriched cellular components (GO_CC). For both panels, cyan and red represent significant enrichment by ToppFun analysis (within the ToppGene Suite) of the 243 downregulated and the 409 upregulated proteins, respectively. FDR: false discovery rate.

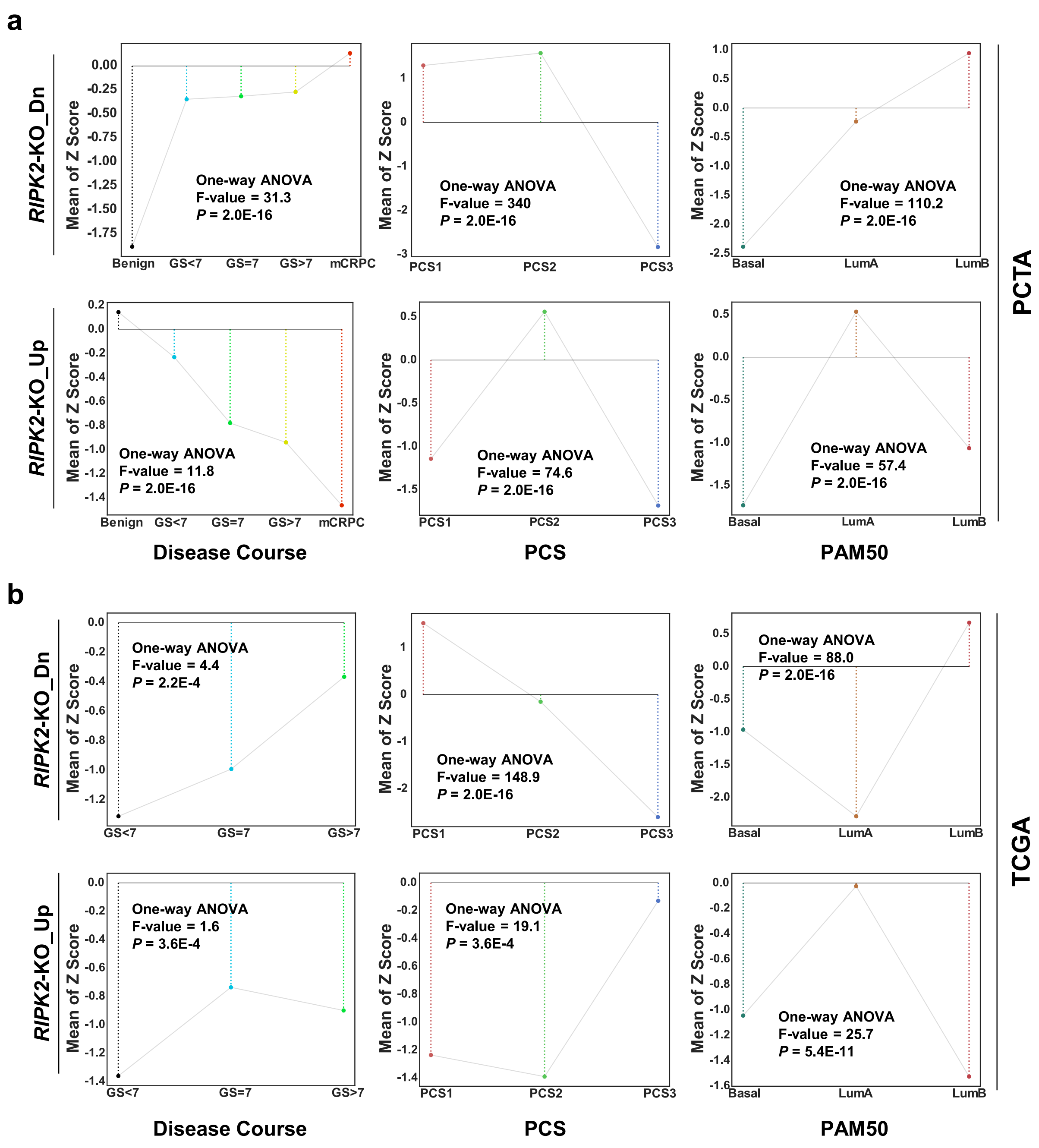

Supplementary Figure 13. Line plots of the mean Z scores of the protein-set downregulated or upregulated by *RIPK2*-KO in PC tissue specimens. a Line plots for the PCTA cohort. **b** Line plots for the TCGA Firehose Legacy cohort. Nominal *p*-values were determined by one-way ANOVA test. RIPK2KO_Dn stands for the 243 proteins downregulated by *RIPK2*-KO, from which RIPK2-induced activity scores were calculated. RIPK2KO_Up represents the 409 proteins upregulated by *RIPK2*-KO, from which RIPK2-repressed activity scores were computed.

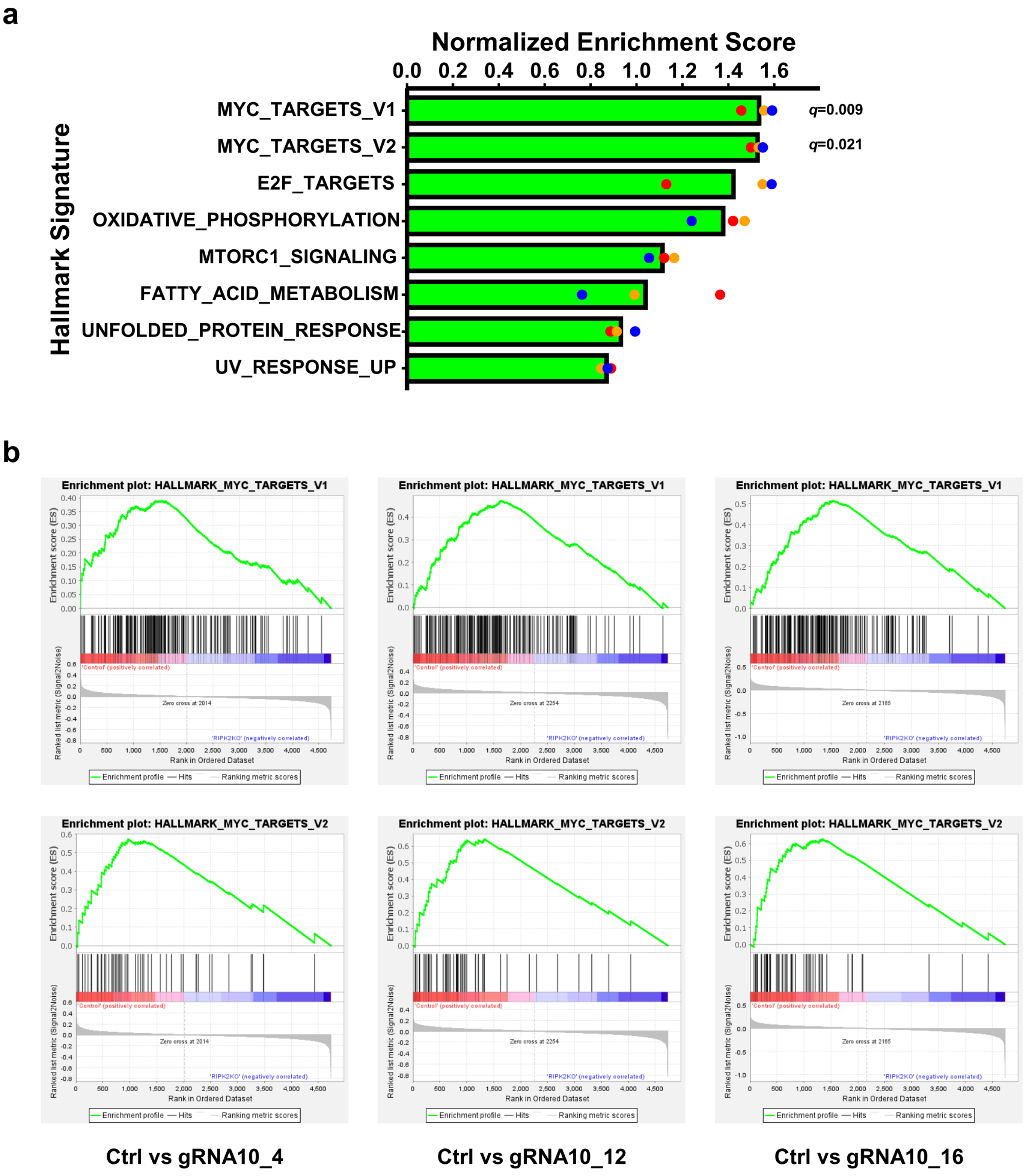

Supplementary Figure 14. MYC gene signatures (V1 and V2) are the most strongly downregulated hallmark signatures by *RIPK2*-KO. a Bar graph of normalized enrichment scores calculated by Gene Set Enrichment Analysis (GSEA) for the top 8 enriched hallmark signatures from the Molecular Signature Database (MSigDB). Red, orange, and blue dots represent the scores derived from *RIPK2*-KO PC3 clones #4, #12, and #16, compared with control PC3 cells. Q values indicate combined *q* values computed by MetaP in R. **b** GSEA plots showing the downregulation of MYC target gene sets V1 (upper) and V2 (lower) in *RIPK2*-KO PC3 clones, compared with control PC3 cells.

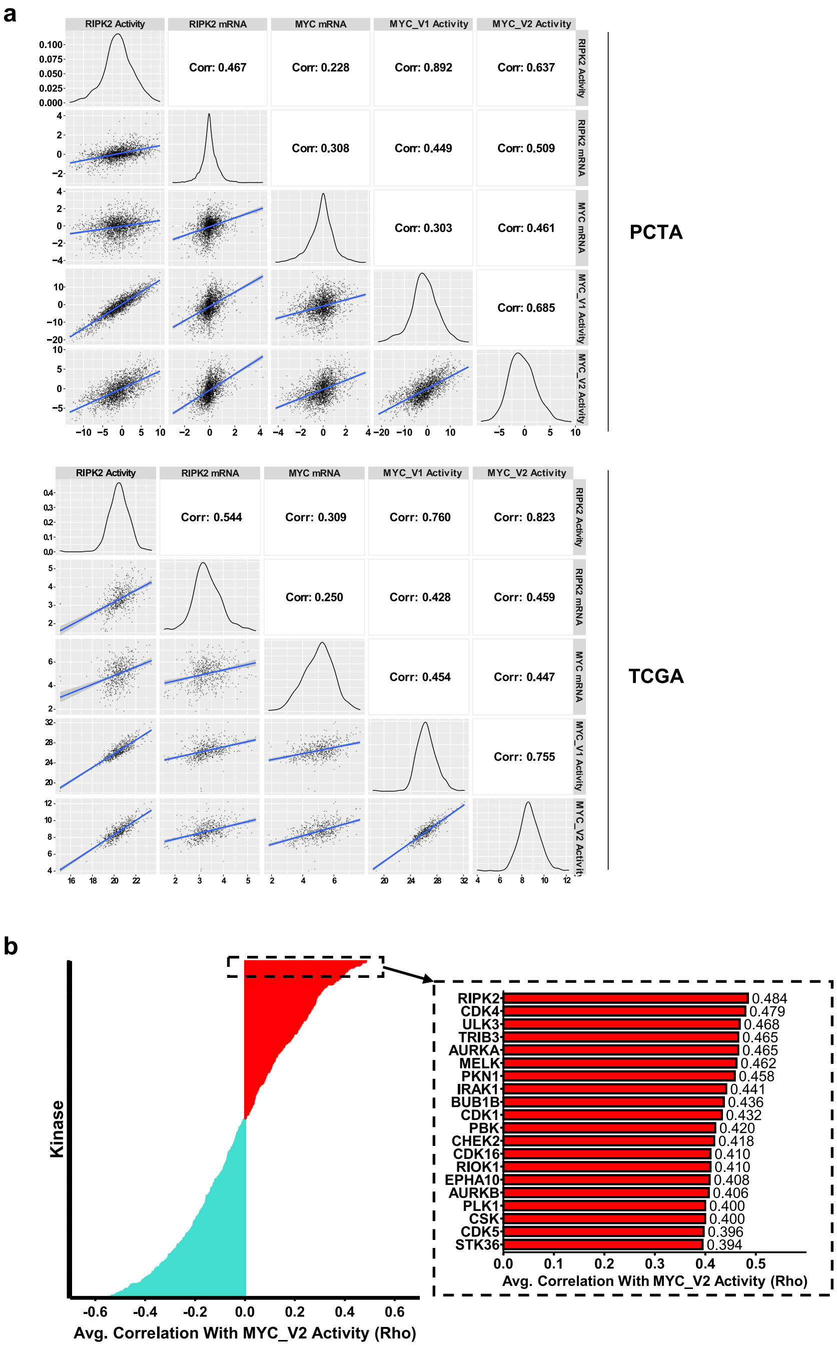

Supplementary Figure 15. *RIPK2*-induced activity scores and *RIPK2* mRNA abundance strongly correlate with *MYC* activity scores in PC tissue specimens. a Correlation matrix showing the correlations between RIPK2 activity scores, *RIPK2* mRNA levels, *MYC* mRNA levels, Hallmark_MYC_Targets_V1 activity scores, and Hallmark_MYC_Targets_V2 activity scores in the PCTA (upper) and the TCGA Firehose Legacy (lower) PC cohorts. Numbers in upper triangle panels indicate Spearman correlation coefficients. Density plots show distribution of numeric variables (*i.e.*, RIPK2 activity scores, *RIPK2* mRNA levels, *MYC* mRNA levels, MYC_V1 activity scores, and MYC_V2 activity scores). Scatter plots present the correlations between each pair of numeric variables. Blue lines indicate linear regression of the data points, and the gray error bands around the blue lines represent the 95% confidence level. **b** Bar graph showing the average rho values of the mRNA levels of each human kinase with Hallmark_MYC_Targets_V2 activity scores in the PCTA and TCGA Firehose Legacy cohorts. The inset shows the average rho values for the top 20 kinases.

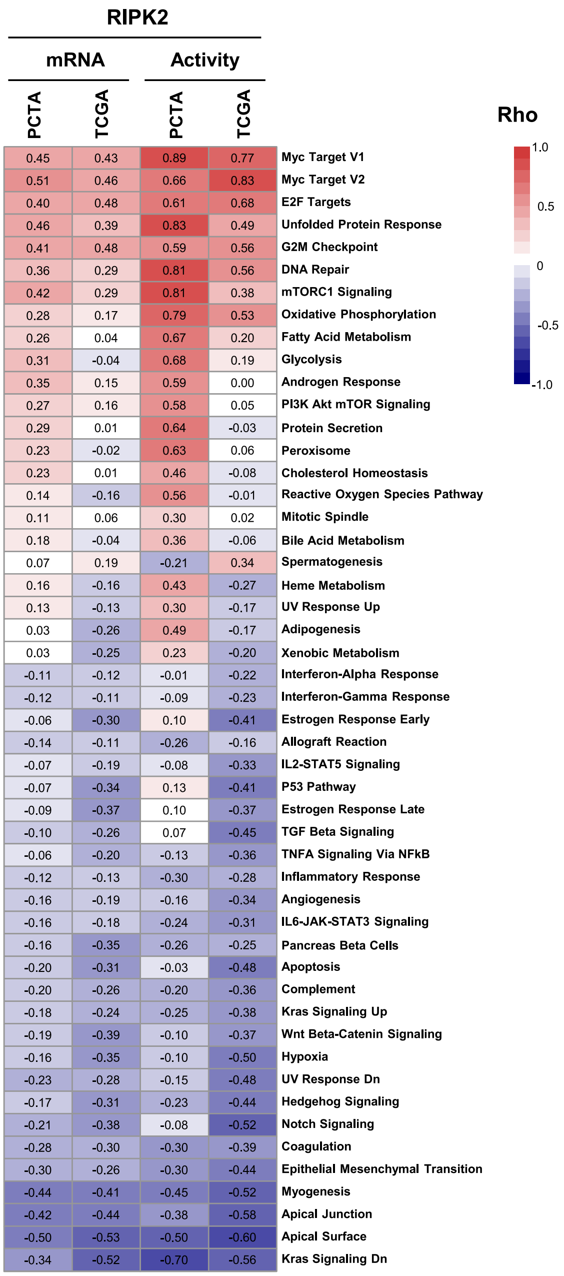

Supplementary Figure 16. Among all MSigDB hallmark gene sets, the *MYC* target gene sets (V1 and V2) are most strongly associated with *RIPK2* mRNA and activity levels in PC tissue specimens. Heatmap of the Spearman's correlation coefficients (rho) of *RIPK2* mRNA and RIPK2-induced activity levels with the activity scores of the 50 MSigDB Hallmark gene sets in the PCTA and TCGA Firehose Legacy cohorts.

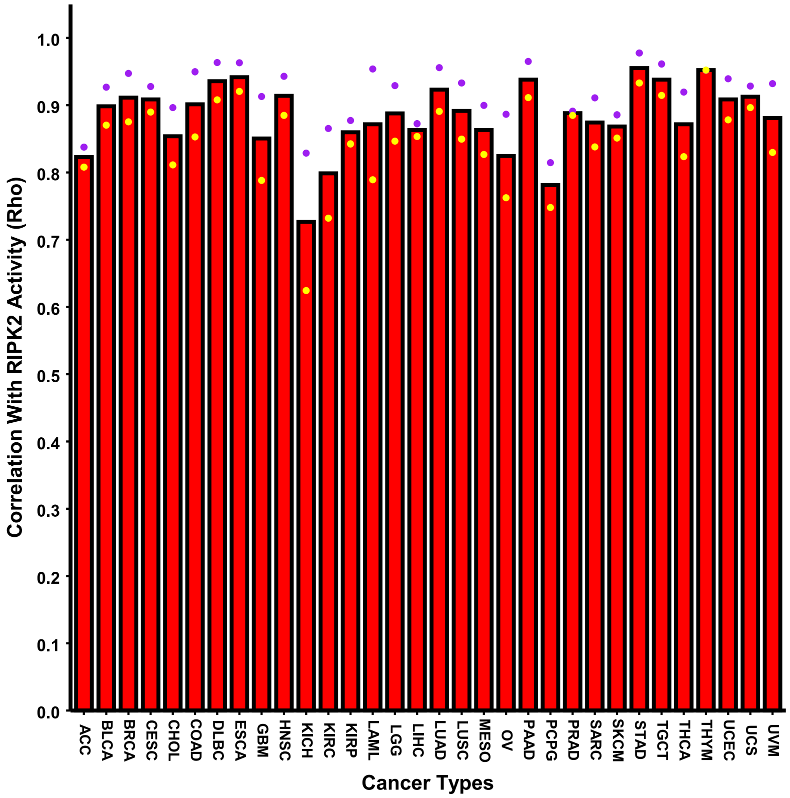

Supplementary Figure 17. *RIPK2*-induced activity scores are strongly associated with *MYC* activity scores across 32 different cancer types. Bar plot of the Spearman's correlation coefficients (rho) between *RIPK2*-induced activity scores and *MYC* activity scores (V1 in purple dots, V2 in yellow dots, and average in bar heights) across 32 TCGA PanCancer Atlas cancer types. ACC: adrenocortical carcinoma; BLCA: bladder urothelial carcinoma; BRCA: breast invasive carcinoma; CHOL: cholangiocarcinoma; COAD: colon adenocarcinoma; DLBC: lymphoid neoplasm diffuse large B-cell lymphoma; ESCA: esophageal carcinoma; GBM: glioblastoma multiforme; HNSC: head and neck squamous cell carcinoma; KICH: kidney chromophobe; KIRC: kidney renal clear cell carcinoma; KIRP: kidney renal papillary cell carcinoma; LAML: acute myeloid leukemia; LGG: brain lower grade glioma; LIHC: liver hepatocellular carcinoma; LUAD: lung adenocarcinoma; LUSC: lung squamous cell carcinoma; MESO: mesothelioma; OV: ovarian serous cystadenocarcinoma; PRAD: prostate adenocarcinoma; SARC: sarcoma; SKCM: skin cutaneous melanoma; STAD: stomach adenocarcinoma; TGCT: testicular germ cell tumors; THCA: thyroid carcinoma; THYM: thymoma; UCEC: uterine corpus endometrial carcinoma; UCS: uterine carcinosarcoma; UVM: uveal melanoma.

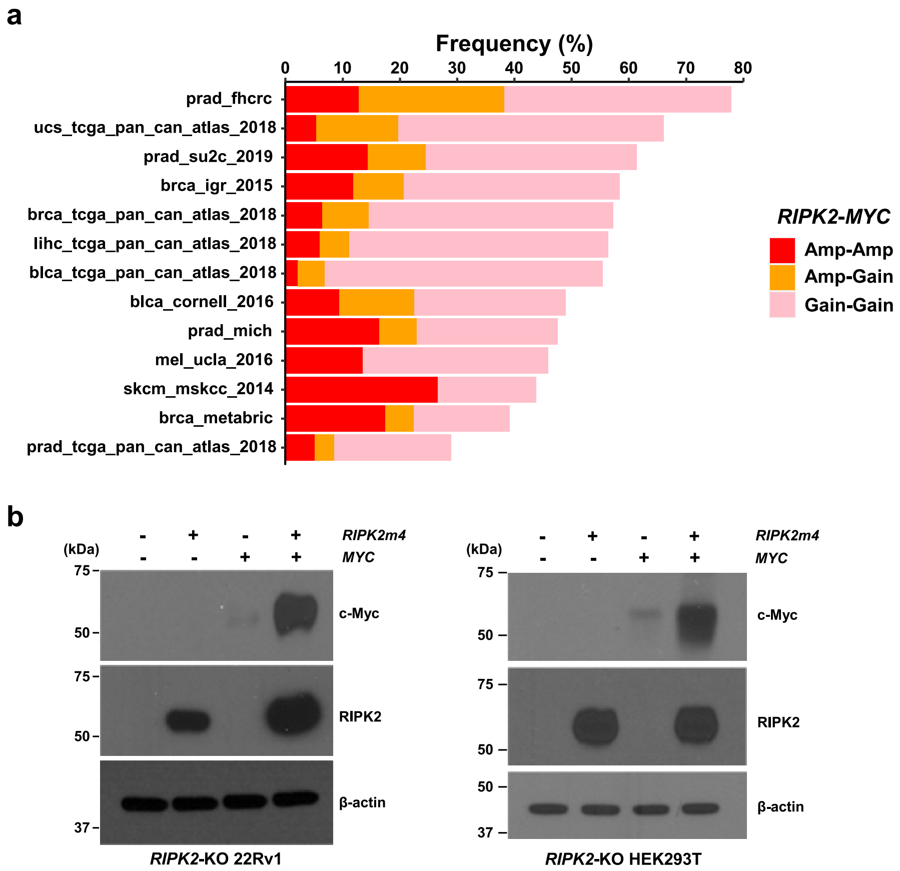

Supplementary Figure 18. The co-amplification/gain of *RIPK2* and *MYC* genes is a frequent event in multiple cancer types and synergistically contributes to c-Myc protein abundance. a Bar plot of the percentages of tumors with *RIPK2* and *MYC* gene co-amplification/gain in different cancer genomics studies (retrieved from the cBioPortal). Different colors show the percentages of tumors with *RIPK2*-amplification and *MYC*-amplification (red), with *RIPK2*-amplification and *MYC*-gain or *RIPK2*-gain and *MYC*-amplification (gold), or with *RIPK2*-gain and *MYC*-gain (pink). prad: prostate adenocarcinoma; ucs: uterine carcinosarcoma; brca: breast invasive carcinoma; lihc: liver hepatocellular carcinoma; blca: bladder urothelial carcinoma; mel: melanoma; skcm: skin cutaneous melanoma. **b** Representative immunoblots of total lysates of *RIPK2*-KO 22Rv1 (left) and HEK293T (right) cells under the indicated conditions. 22Rv1 cells were transfected with 1.0 µg *RIPK2m4* and/or 1.0 µg *MYC* plasmids; HEK293T cells were transfected with 0.5 µg *RIPK2m4* and/or 0.5 µg *MYC* plasmids. β-actin was used as a loading control.

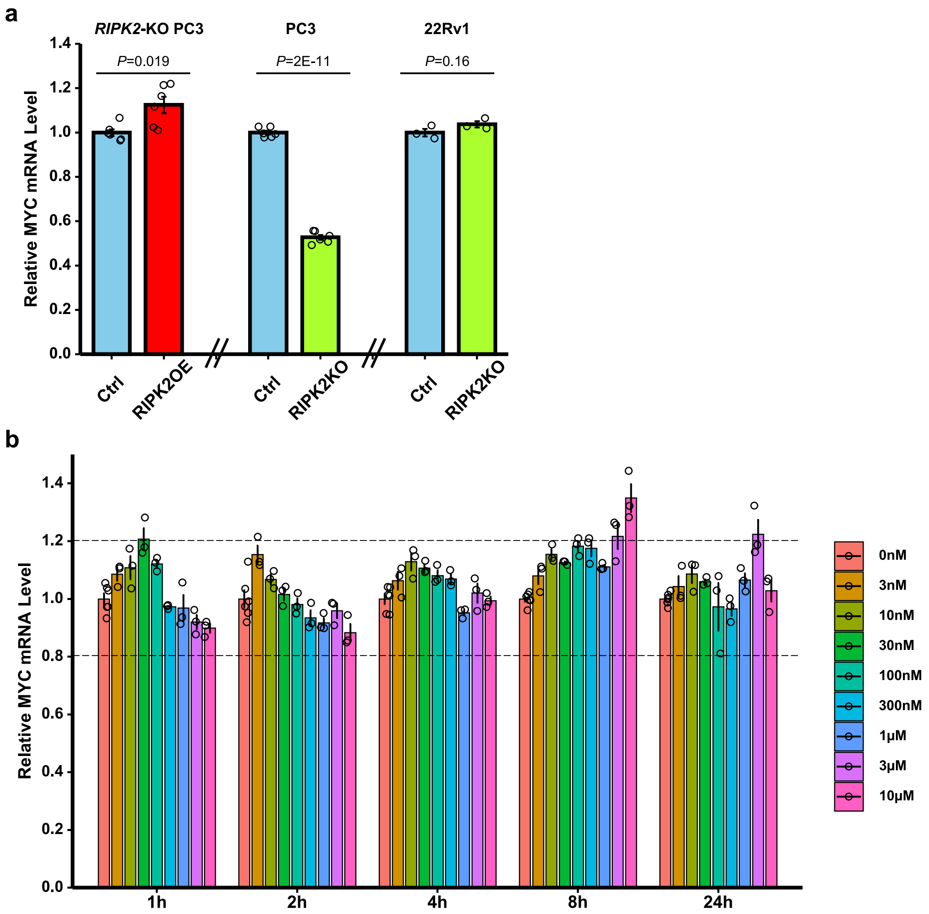

Supplementary Figure 19. The regulation of c-Myc by RIPK2 is mainly not at the transcriptional level. a Bar plot of *MYC* mRNA level changes (normalized to *GAPDH*) caused by transient overexpression of *RIPK2m4* in *RIPK2*-KO PC3 cells (n = 6 biologically independent samples per group), *RIPK2*-KO in PC3 cells (n = 6 biologically independent samples per group), or *RIPK2*-KO in 22Rv1 cells (n = 3 biologically independent samples per group). Data are Mean ± SD. Nominal *p*-values were determined by unpaired two-tailed Student's *t*-test. **b** Bar plot of *MYC* mRNA level changes (normalized to *GAPDH*) in PC3 cells in response to GSK583 at varied doses for different periods of time (n = 3 biologically independent samples per group, with the exception of n = 6 biologically independent samples in 0 nM groups). Data are Mean ± SD.

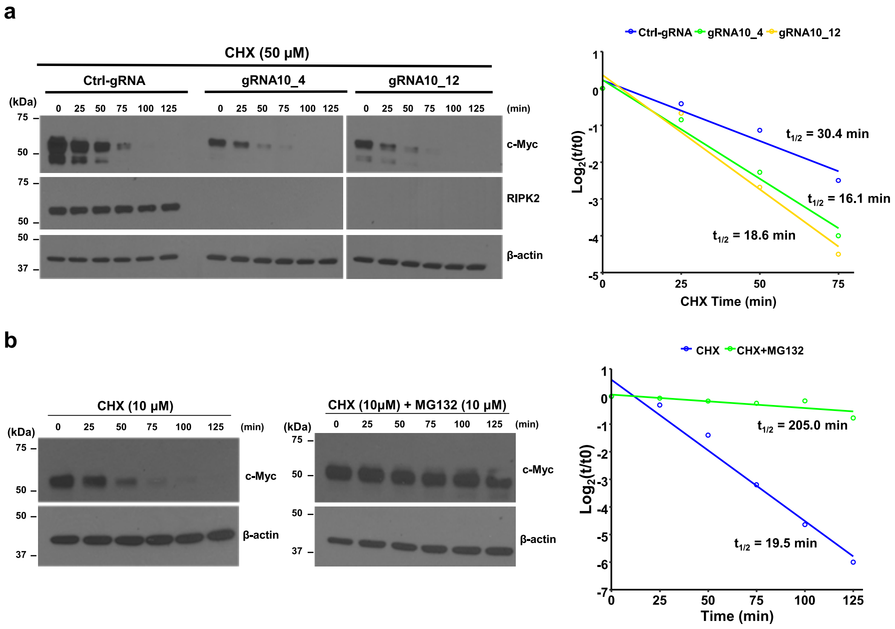

Supplementary Figure 20. Genetic knockout of *RIPK2* destabilizes the c-Myc protein by promoting its proteasomal degradation. a Representative immunoblots (left) and quantification (right) of c-Myc protein levels in total lysates of *RIPK2*-KO PC3 single-cell clones #4 and #12, compared with control PC3 cells, in response to cycloheximide (CHX) treatment (n = 4 biologically independent samples per group). **b** Representative immunoblots (left) and quantification (right) of c-Myc protein levels in total lysates of PC3 cells treated with DMSO or MG132, in the presence of CHX treatment. Experiments were repeated twice independently with similar results (panel b).

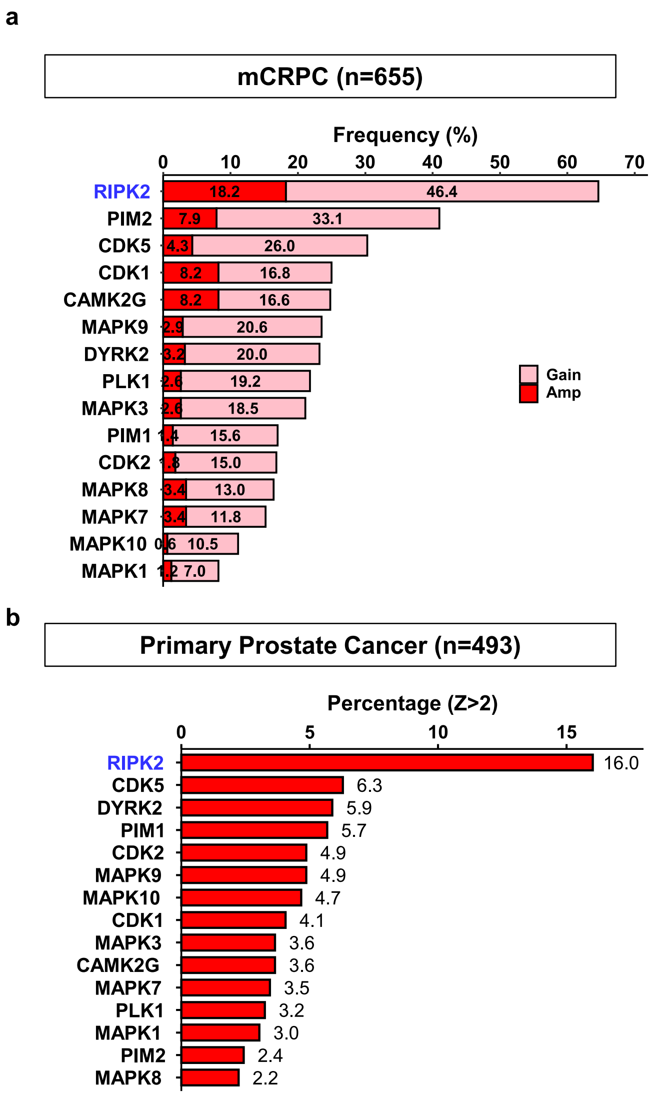

Supplementary Figure 21. The genetic alteration of *RIPK2* is more frequent than that of any known direct c-Myc-S62 kinases in PC tissue specimens. a Bar plot of the percentages of mCRPC tumors (n = 655) with gene amplification/gain. Numbers in bars indicate percentages. **b** Bar plot of the frequency of gene overexpression (Z > 2 relative to diploid samples) in the TCGA PanCancer Atlas primary PC cohort (n = 493). Numbers to the right of bars indicate percentages.

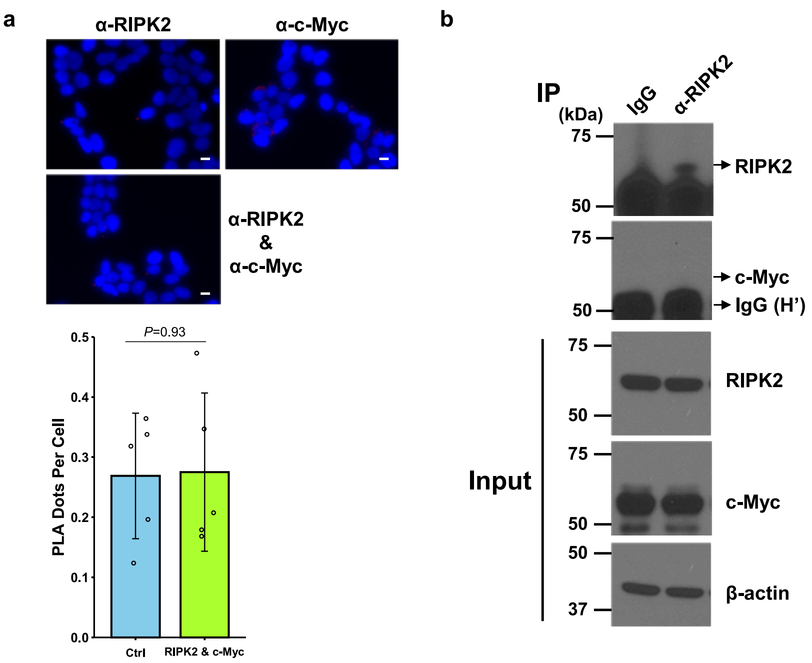

Supplementary Figure 22. RIPK2 does not bind to c-Myc under physiological condition. a Representative PLA (proximity ligation assay) images (upper) and quantification (lower) of endogenous RIPK2 and c-Myc proteins in parental HEK293T cells. Scale bars, 10 μm. Data are Mean ± SD (n = 5 biologically independent samples per group). Nominal *p*-value was determined by unpaired two-tailed Student's *t*-test. **b** Representative immunoblots of RIPK2 and c-Myc in RIPK2 protein complexes immunoprecipitated from parental PC3 cells (upper) or in PC3 total lysates (lower).

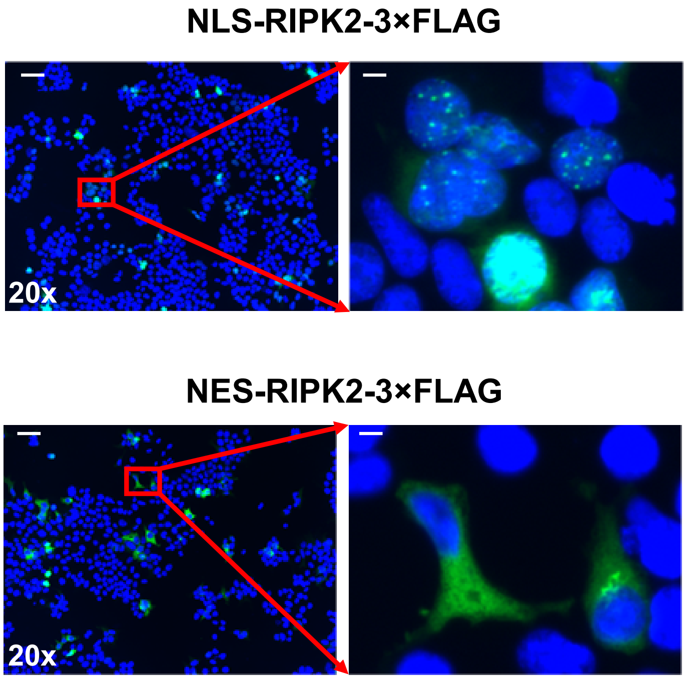

Supplementary Figure 23. NLS and NES specifically target RIPK2 into the nuclei and the cytoplasm, respectively. *RIPK2*-KO HEK293T cells were transiently transfected with N-terminally NLS-tagged (upper) or NES-tagged (lower) RIPK2m4, both of which were C-terminally tagged with a 3×FLAG tag. An anti-FLAG antibody was used for immunofluorescence imaging. Scale bars, 50 μm (left) or 5 μm (right). Experiments were repeated twice independently with similar results.

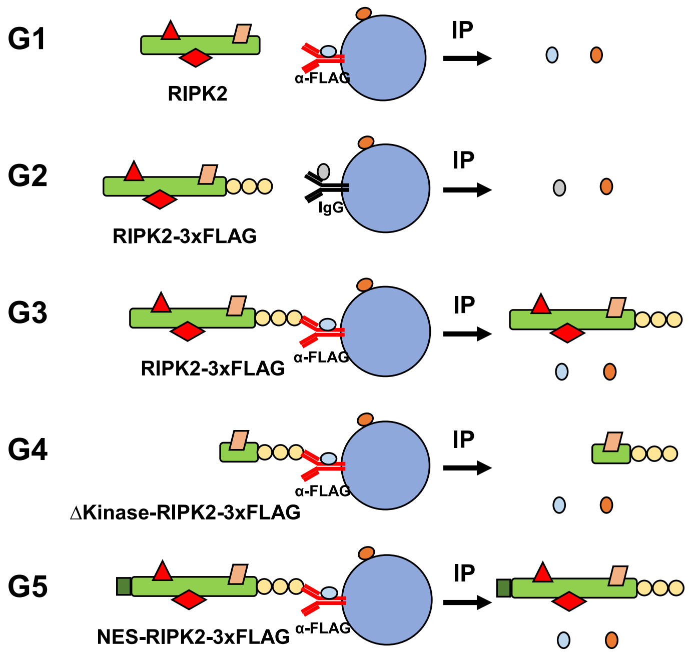

Supplementary Figure 24. Illustration of RIPK2 complexes and predicted immunoprecipitation products. Green rounded rectangle: full-length RIPK2 (long) or Δkinase-RIPK2 (short); three yellow circles: 3×FLAG; red triangles and diamonds: proteins specifically bound to the kinase domain and no other regions of RIPK2; deep green square: NES. WT: wild-type; Δkinase: deletion of the kinase domain; NES: nuclear export signal. The G3 and G5 are experimental groups, whereas the G1, G2, and G4 groups are control groups.

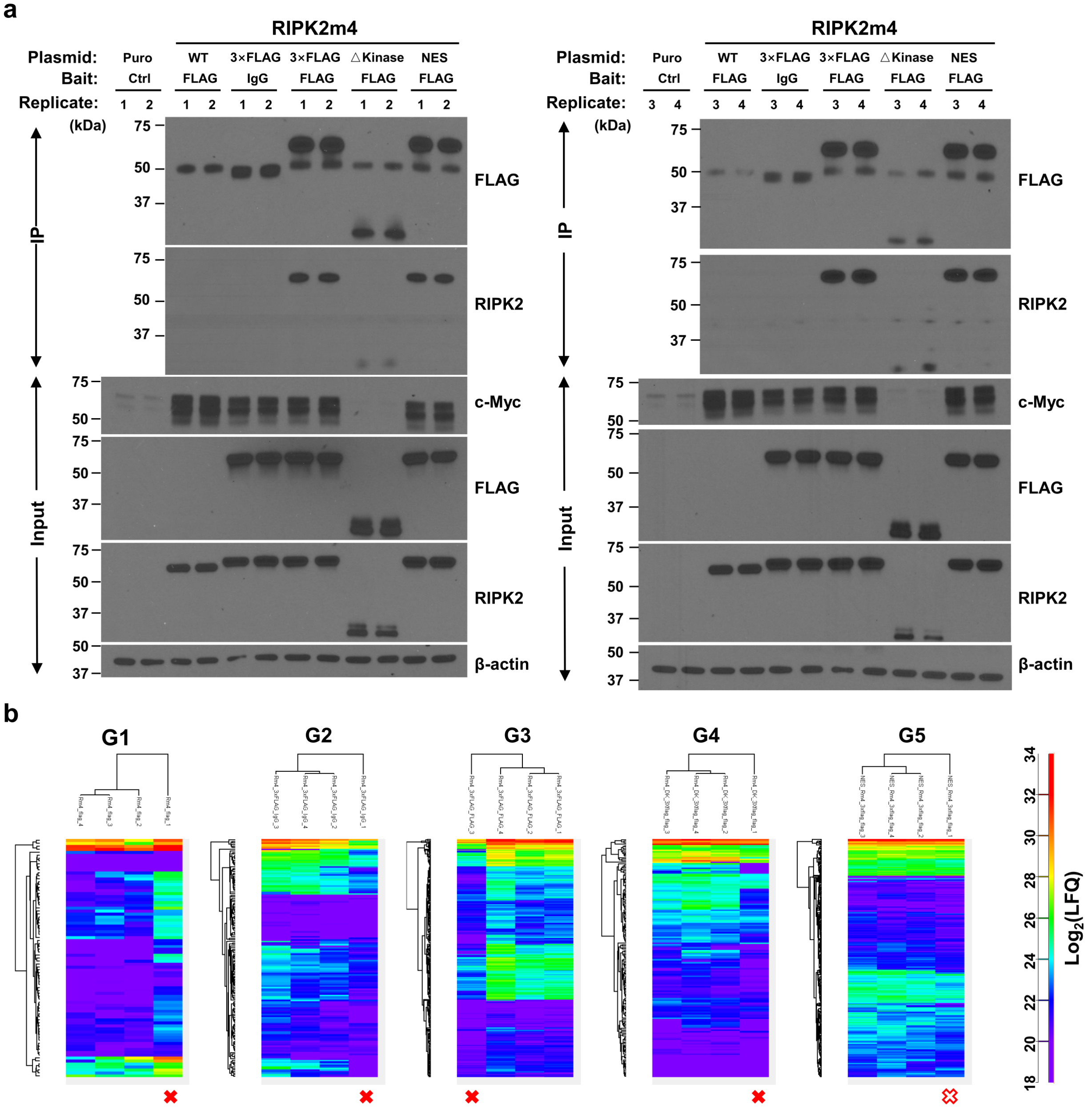

Supplementary Figure 25. Quality assessment of the immunoprecipitation samples. a Immunoblots of the indicated proteins in the four IP biological replicates. *RIPK2*-KO HEK293T cells were transiently transfected with the indicated forms of plasmids, and total lysates were subjected to IP using antibodies against the indicated baits. IP products and total lysates (input) were analyzed by immunoblots to probe the indicated proteins. **b** Unsupervised clustering of the $\log_2$(LFQ) values to detect outlier samples (labeled with ×) in the five IP groups (G1-G5). IP products were analyzed by in-gel digestion coupled with mass spectrometric analysis (GeLC-MS/MS). The label-free quantification (LFQ) values were log2-transformed and clustered. Outlier samples were detected by visually inspecting the hierarchical cluster heatmaps.

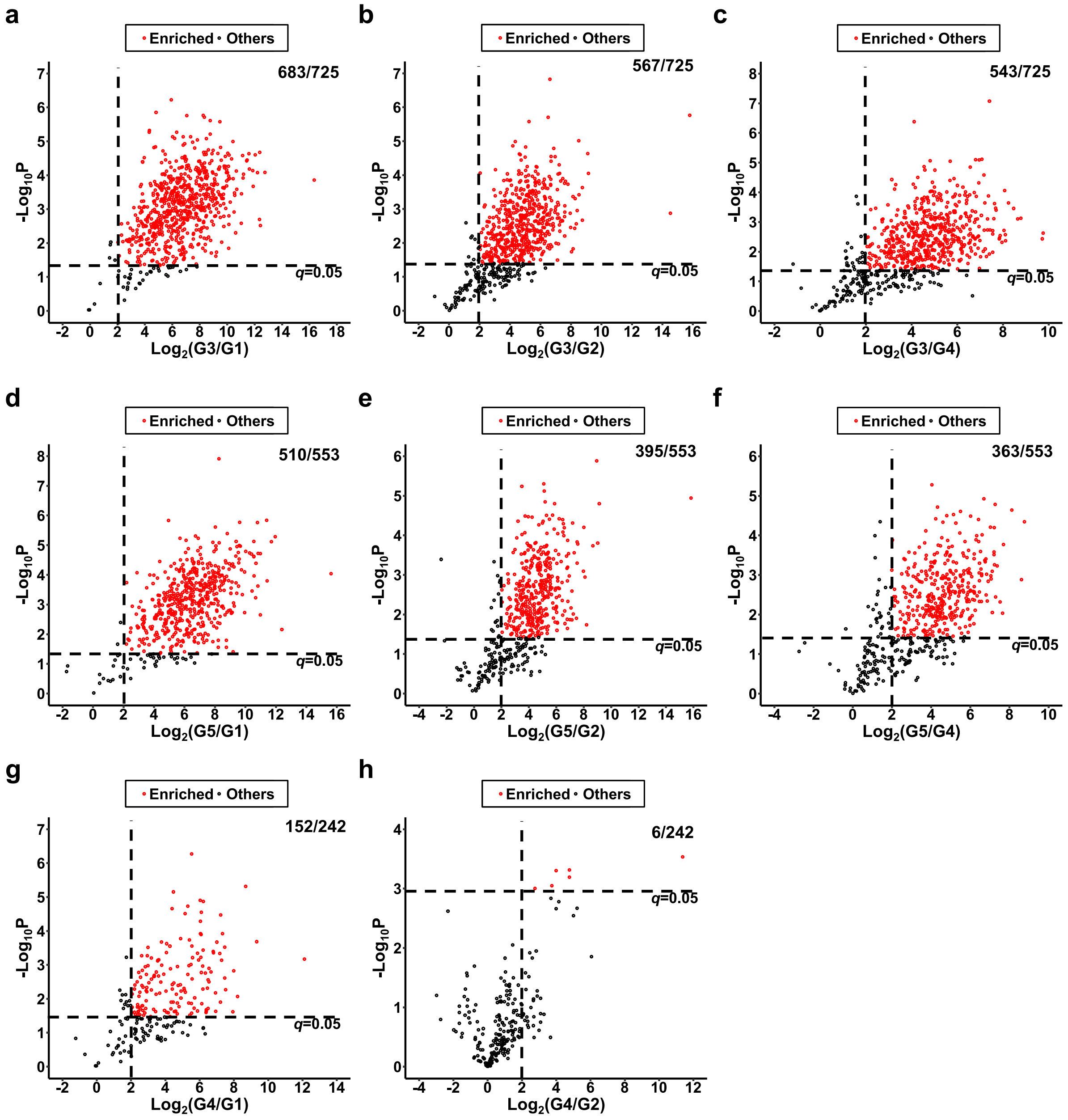

Supplementary Figure 26. Identification of significantly enriched proteins in eight IP-MS comparisons. a-h Volcano plots showing the $\log_2$-transformed protein ratios versus the $-\log_{10}(P)$ values following quantitative comparison of (a) G3 *vs.* G1, (b) G3 *vs.* G2, (c) G3 *vs.* G4, (d) G5 *vs.* G1, (e) G5 *vs.* G2, (f) G5 *vs.* G4, (g) G4 *vs.* G1, and (h) G4 *vs.* G2. Each dot represents a protein group; red dots represent significantly enriched protein groups ($q < 0.05$ and $\log_2$Ratio > 2). Fractions in the upper right corners represent the numbers of significantly enriched proteins divided by those of total quantified proteins.

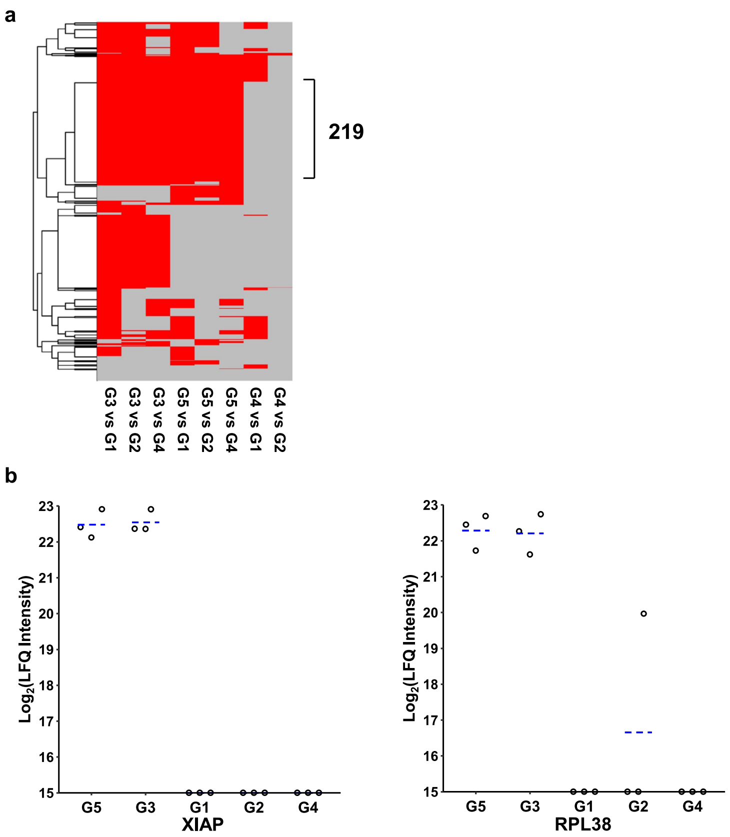

Supplementary Figure 27. Identification of candidate proteins associated with the kinase domain of cytoplasmic RIPK2. a Unsupervised clustering of protein groups in each of the eight comparisons to identify candidate proteins associated with the kinase domain and no other regions of cytoplasmic RIPK2. Red represents significant enrichment ($q < 0.05$ and $log_2Ratio > 2$); gray represents insignificant enrichment or no quantification. **b** Swarm plot of log_2(LFQ) values of XIAP (left) and RPL38 (right), two known RIPK2-interacting proteins, in the five IP-MS groups (n = 3 biologically independent samples per group).

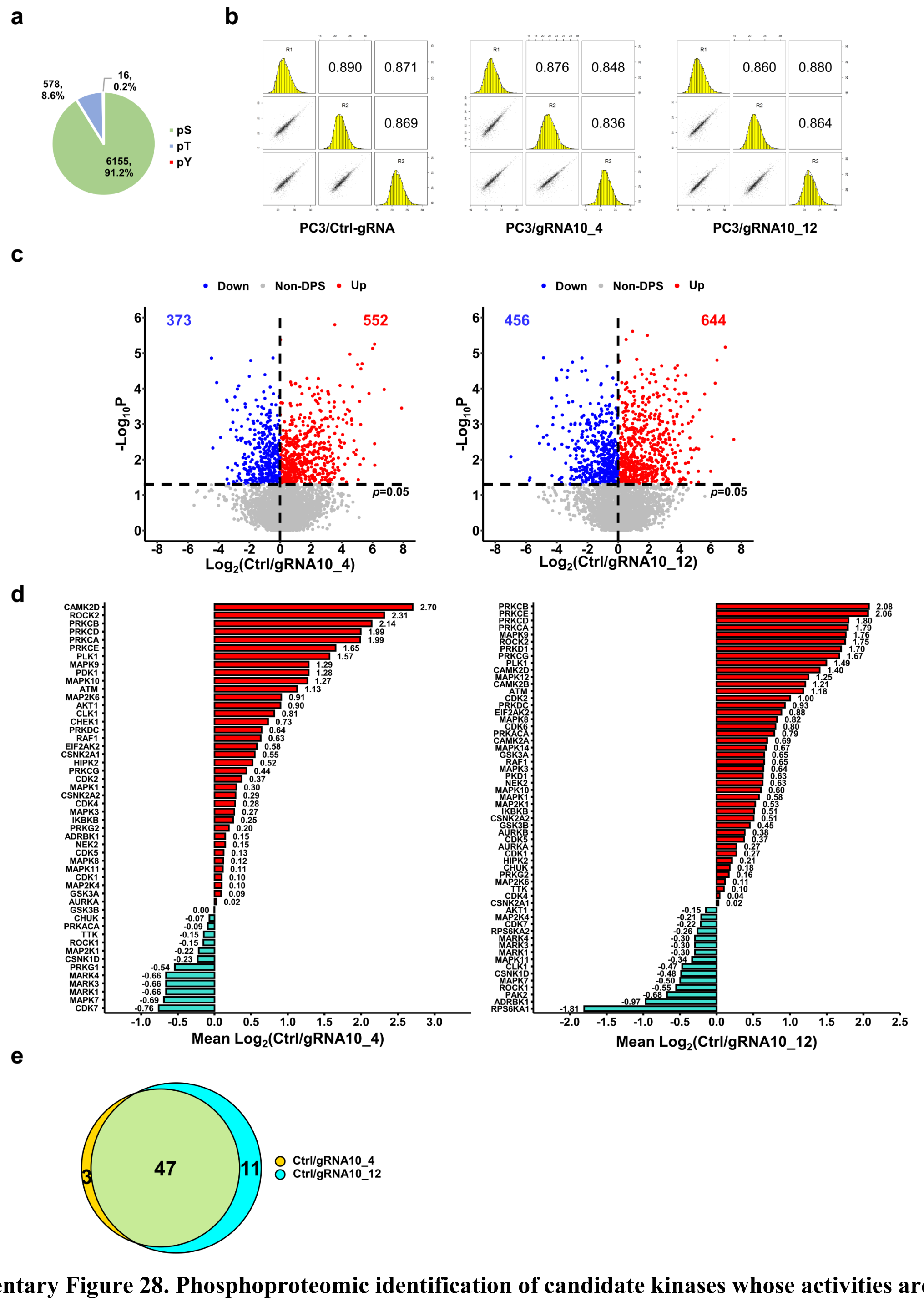

Supplementary Figure 28. Phosphoproteomic identification of candidate kinases whose activities are regulated by RIPK2. a Pie chart of the 6,749 identified phosphoserine (pS), phosphothreonine (pT), and phosphotyrosine (pY) sites (FDR < 1% and localization probability > 0.75). **b** Quality assessment of the phosphoproteomics data. Numbers in boxes: Pearson correlation coefficients between replicates; histograms: distribution of $\log_2$(LFQ) values in each replicate; R1-R3: biological replicates #1-#3; scatter plots: distribution of $\log_2$(LFQ) values of phosphosites in two different replicates. **c** Volcano plots of the phosphorylation levels of all quantified phosphosites in control PC3 cells relative to *RIPK2*-KO PC3 clone#4 (left) or clone#12 (right). Non-DPS, non-differentially phosphorylated sites. **d** Bar plots of the inferred activities of candidate kinases downstream of RIPK2 in control PC3 cells relative to *RIPK2*-KO PC3 clone#4 (left) or clone#12 (right). **e** Venn diagram of candidate RIPK2-regulated kinases identified from control PC3 cells in comparison to the two *RIPK2*-KO PC3 single-cell clones.

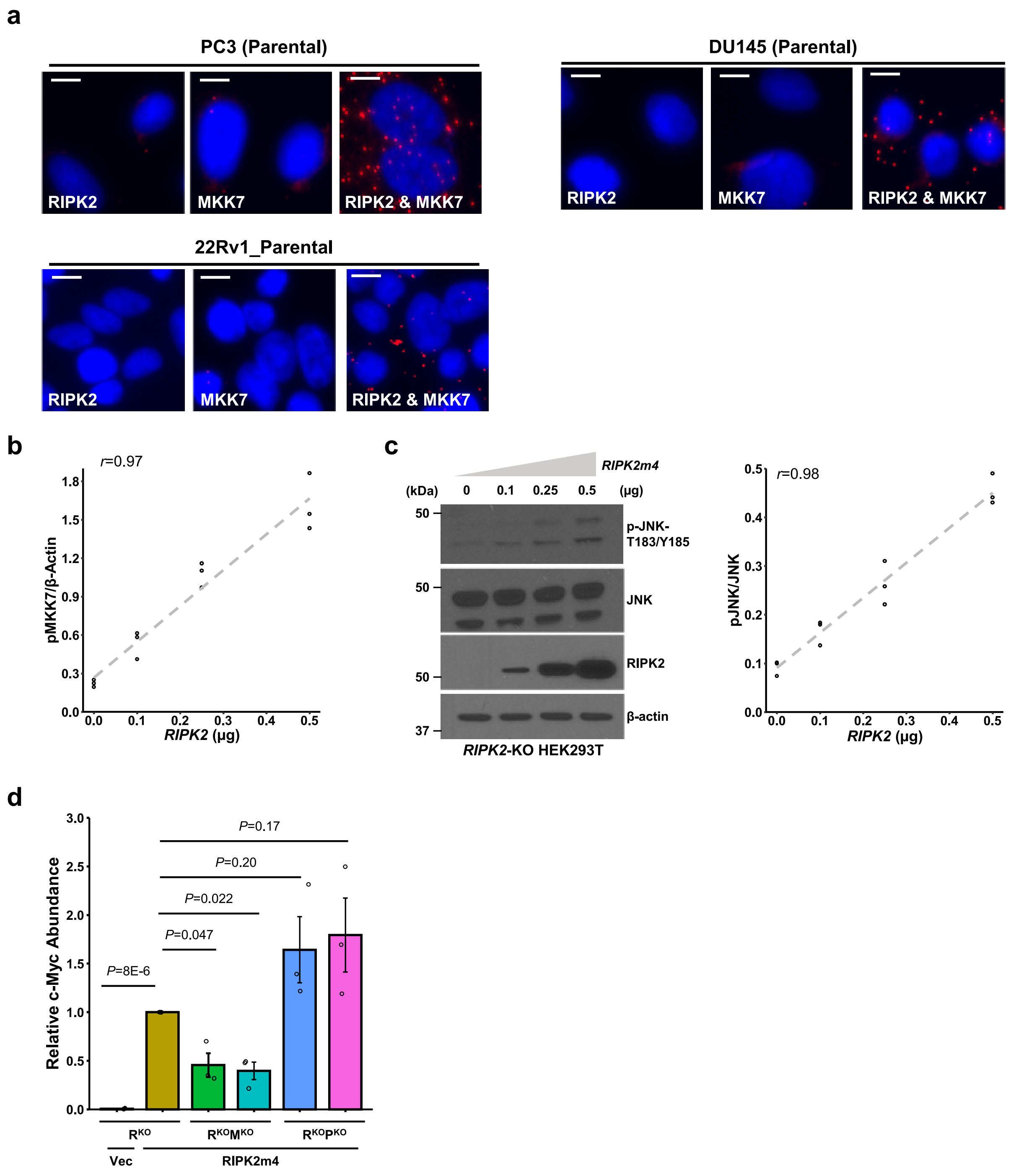

Supplementary Figure 29. RIPK2 binds to and activates MKK7, a major mediator of RIPK2 regulation of c-Myc. a Representative PLA images of endogenous RIPK2 and MKK7 proteins in parental PC cells. Scale bars, 10 µm. **b** Scatter plot of the pMKK7-S271 levels in *RIPK2*-KO HEK293T cells transiently transfected with different doses of the *RIPK2m4* plasmid (n = 3 biologically independent samples per group). **c** Representative immunoblots (left) and scatter plot (right) of the pJNK-T183/Y185 levels in *RIPK2*-KO HEK293T cells transiently transfected with different doses of the *RIPK2m4* plasmid (n = 3 biologically independent samples per group). **d** Bar plot of the relative c-Myc protein abundance in HEK293T cells under the indicated conditions. Nominal *p*-values were determined by unpaired two-tailed Student's *t*-test. Data are mean ± SEM (n = 3 biologically independent samples per group).

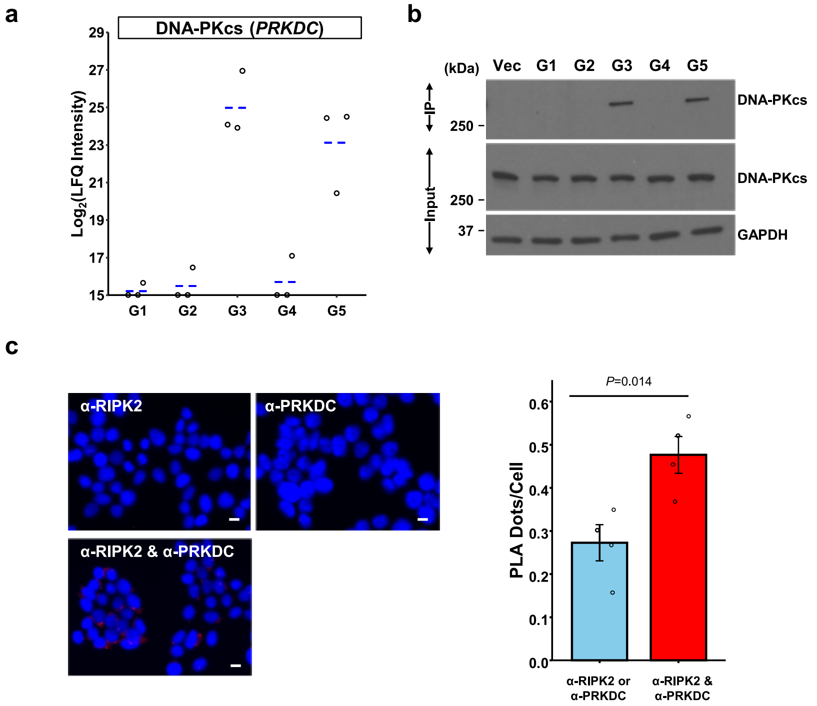

Supplementary Figure 30. DNA-PKcs encoded by *PRKDC* is a RIPK2-interacting protein. a Swarm plot of $\log_2$(LFQ) values of DNA-PKcs (encoded by *PRKDC*) in the five IP-MS groups (n = 3 biologically independent samples per group). **b** Representative immunoblots of DNA-PKcs (*PRKDC*) in immunoprecipitated RIPK2 protein complexes or in total lysates corresponding to the five IP-MS groups (G1-G5). Experiments were repeated twice independently with similar results. **c** Cellular images (left) and quantification (right) of the PLA detecting the association of endogenous RIPK2 and DNA-PKcs (*PRKDC*) in parental HEK293T cells (n = 4 biologically independent samples per group). Scale bars, 10 µm. Nominal *p*-value was determined by unpaired two-tailed Student's *t*-test. Data are mean ± SEM.

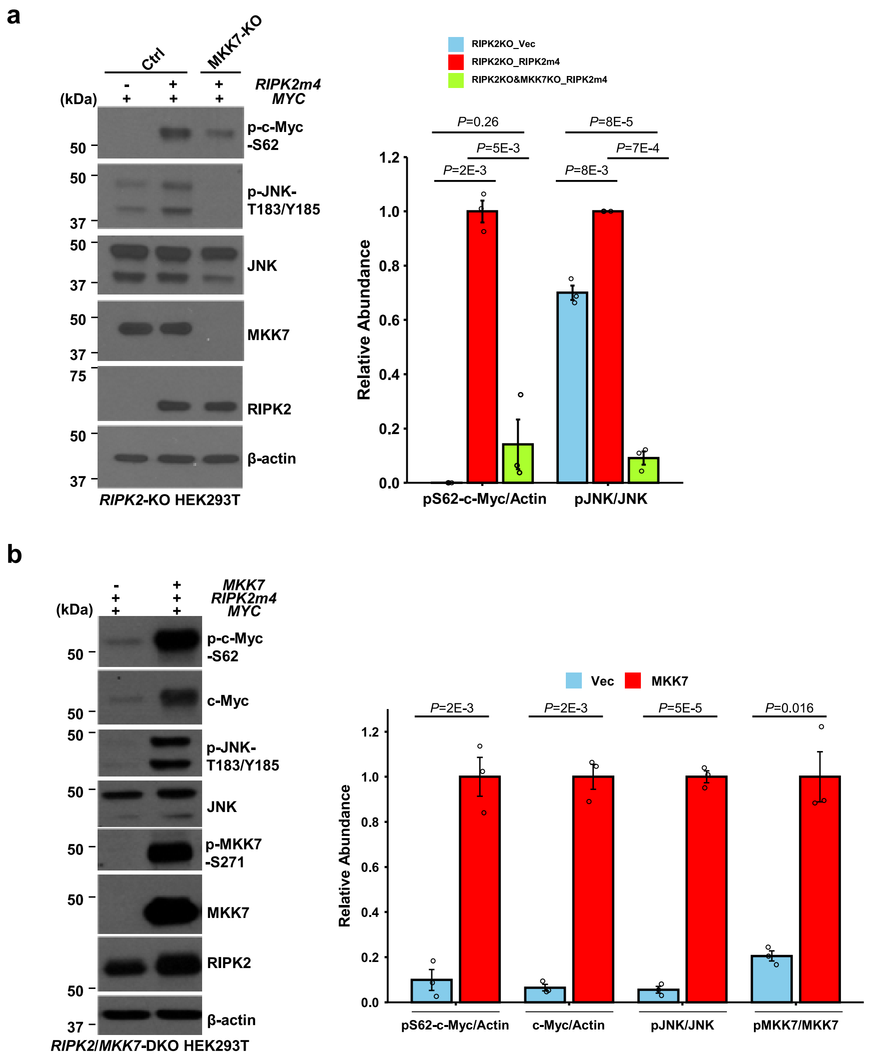

Supplementary Figure 31. MKK7 mediates RIPK2-induced c-Myc and JNK phosphorylation. a Representative immunoblotting images of the indicated proteins (left) and bar graph of relative protein abundance or phosphorylation levels (right) in HEK293T cells under the indicated conditions (n = 3 biologically independent samples per group). **b** Representative immunoblotting images of the indicated proteins (left) and bar graph of relative protein abundance or phosphorylation levels (right) in HEK293T cells with *RIPK2*- and *MKK7*-double knockout (*RIPK2*/*MKK7*-DKO), which were transiently transfected with the *RIPK2m4* plasmid and vector or *MKK7* (n = 3 biologically independent samples per group). Nominal *p*-values were determined by unpaired two-tailed Student's *t*-test. Data are Mean ± SEM.

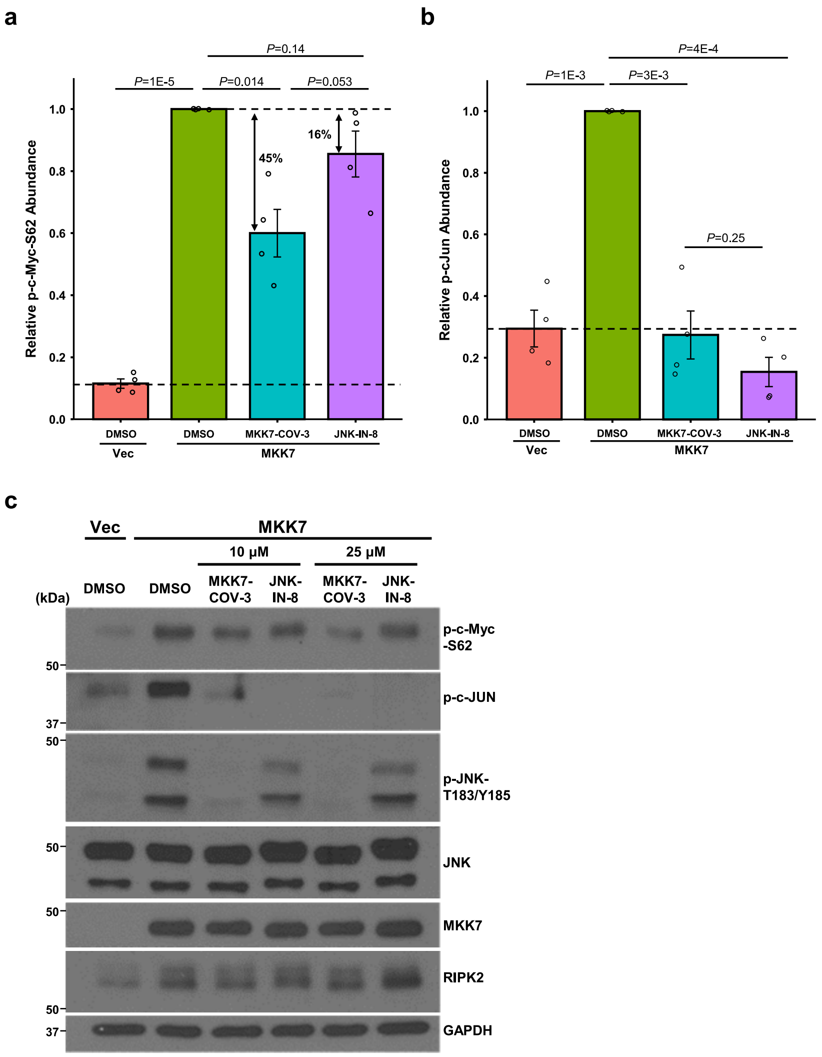

Supplementary Figure 32. JNK only mediates a subset of RIPK2/MKK7 phosphorylation of c-Myc-S62. *RIPK2/MKK7*-DKO HEK293T cells were transiently transfected with 0.5 µg *RIPK2m4,* 0.5 µg *MYC,* and 0.1 µg *MAP2K7* or vector plasmids. The cells were then treated with the MKK7 inhibitor MKK7-COV-3, the JNK inhibitor JNK-IN-8, or vehicle control. **a** Bar graph of relative p-c-Myc-S62 abundance under the indicated conditions. MKK7-COV-3, 25 µM; JNK-IN-8, 1 µM; 2-h treatment. **b** Bar graph of relative p-c-Jun abundance under the indicated conditions. MKK7-COV-3: 25 µM; JNK-IN-8: 1 µM; 2-h treatment. **c** Representative immunoblots of the indicated proteins under the indicated conditions. Experiments were repeated twice independently with similar results. Nominal *p*-values were determined by unpaired two-tailed Student's *t*-test (panels a and b). Data are Mean ± SEM (n = 4 biologically independent samples per group) (panels a and b).

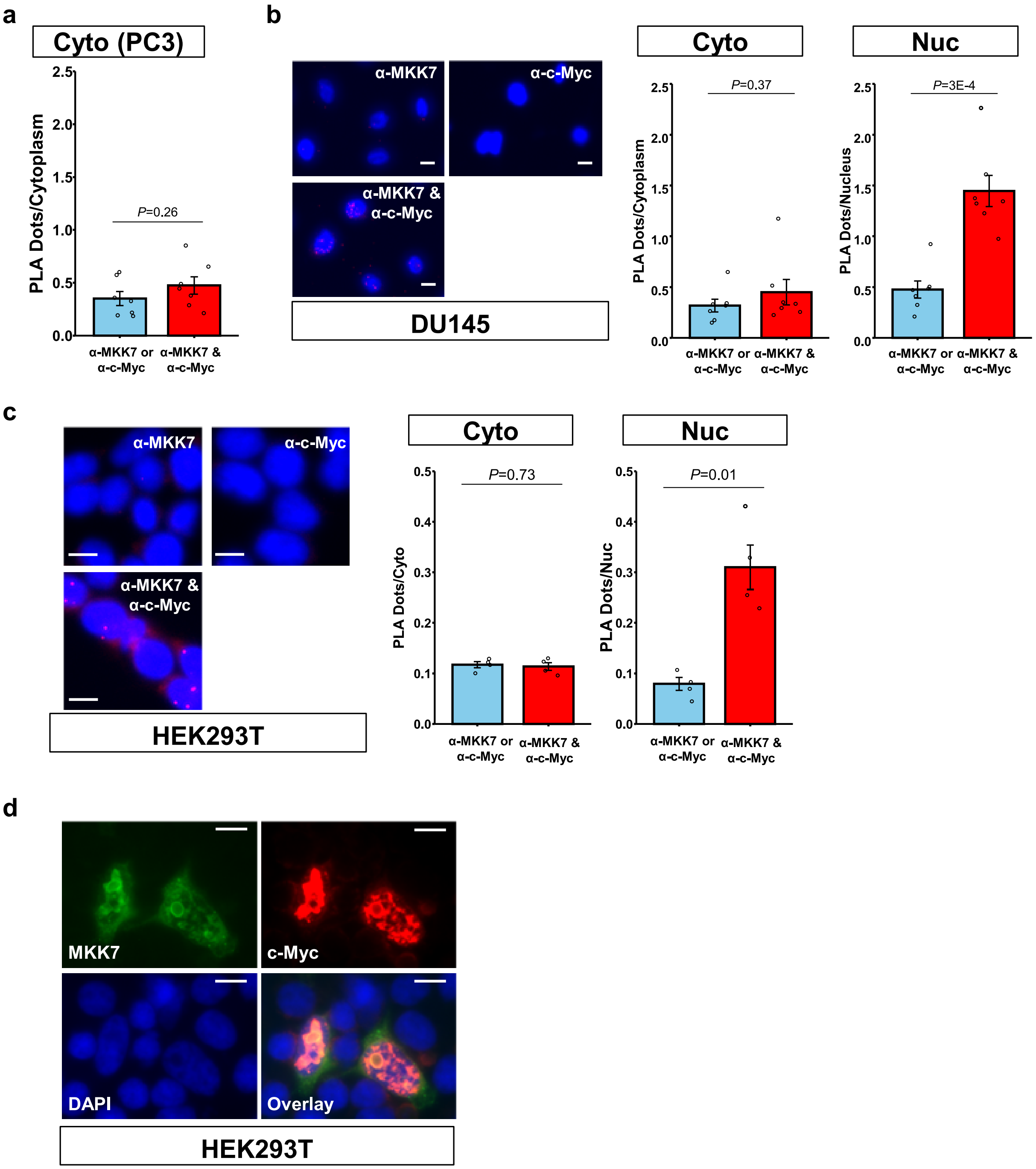

Supplementary Figure 33. MKK7 associates with c-Myc in the nuclei. a Quantification of PLA analysis of endogenous MKK7 and c-Myc proteins in the cytoplasm of PC3 cells (n = 7 biologically independent samples per group). **b** Cellular images (left) and quantification (right) of PLA detecting the association of endogenous MKK7 and c-Myc proteins in DU145 cells (n = 7 biologically independent samples per group). Scale bars, 10 µm. **c** Cellular images (left) and quantification (right) of PLA detecting the association of endogenous MKK7 and c-Myc proteins in HEK293T cells (n = 7 biologically independent samples per group). Scale bars, 10 µm. **d** Representative immunofluorescence images of MKK7 and c-Myc in *RIPK2/MKK7*-DKO HEK293T cells, which were transiently transfected with *RIPK2m4*, *MKK7-His*, and *MYC* plasmids. Experiments were repeated three times independently with similar results. Scale bars, 10 µm. Nominal *p*-values were determined by unpaired two-tailed Student's *t*-test (panels a-c). Data are mean ± SEM (panels a-c).

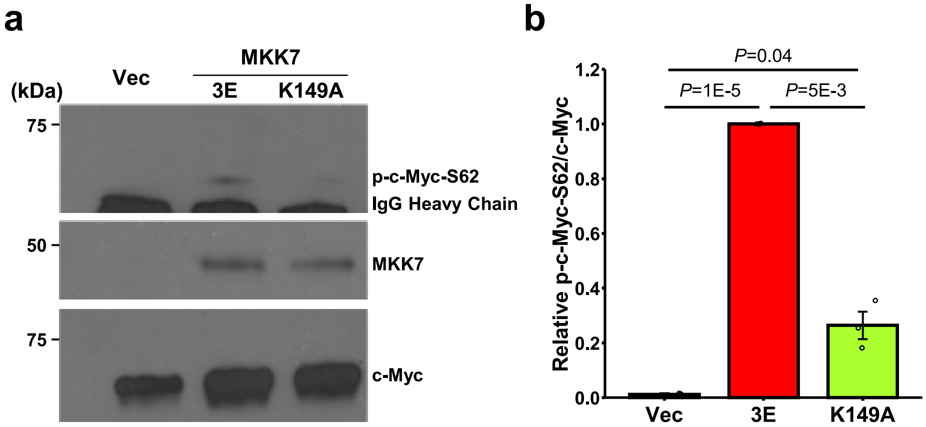

Supplementary Figure 34. Kinase-dead MKK7-K149A is much less efficient than constitutively active MKK7-3E (S271E, T275E, S277E) in phosphorylating c-Myc-S62 *in vitro*. a Representative immunoblots of the indicated proteins after *in vitro* kinase assay reactions. *RIPK2/MKK7*-DKO HEK293T cells were transiently transfected with *MKK7-3E*, *MKK7-K149A*, or vector control. Whole cell lysates were subjected to immunoprecipitation using an anti-MKK7 antibody. Enriched proteins were incubated with recombinant c-Myc for *in vitro* kinase assays. Experiments were repeated three times independently with similar results. **b** Quantification of relative c-Myc-S62 phosphorylation levels under the indicated conditions (n = 3 biologically independent samples per group). Data are shown as Mean ± SEM. Nominal *p*-values were determined by two-tailed Student's *t*-test.

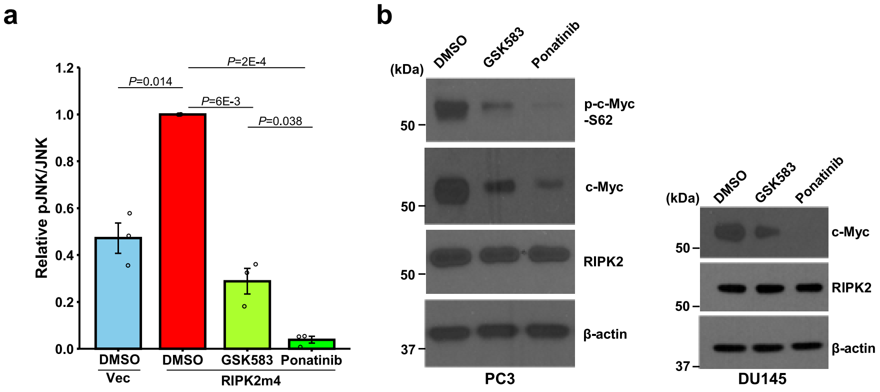

Supplementary Figure 35. Pharmacological inhibition of RIPK2 reduces p-JNK, p-c-Myc-S62, and c-Myc levels. a Bar plot of the relative phosphorylation levels of JNK in *RIPK2*-KO HEK293T cells under the indicated conditions (n = 3 biologically independent samples per group). Nominal *p*-values were determined by unpaired two-tailed Student's *t*-test. Data are mean ± SEM. **b** Representative immunoblots of the indicated proteins in total lysates of PC3 (left) or DU145 (right) cells, which were treated with vehicle control, 10 μM GSK583, or 5 μM ponatinib for 2 h. Experiments were repeated three times independently with similar results.

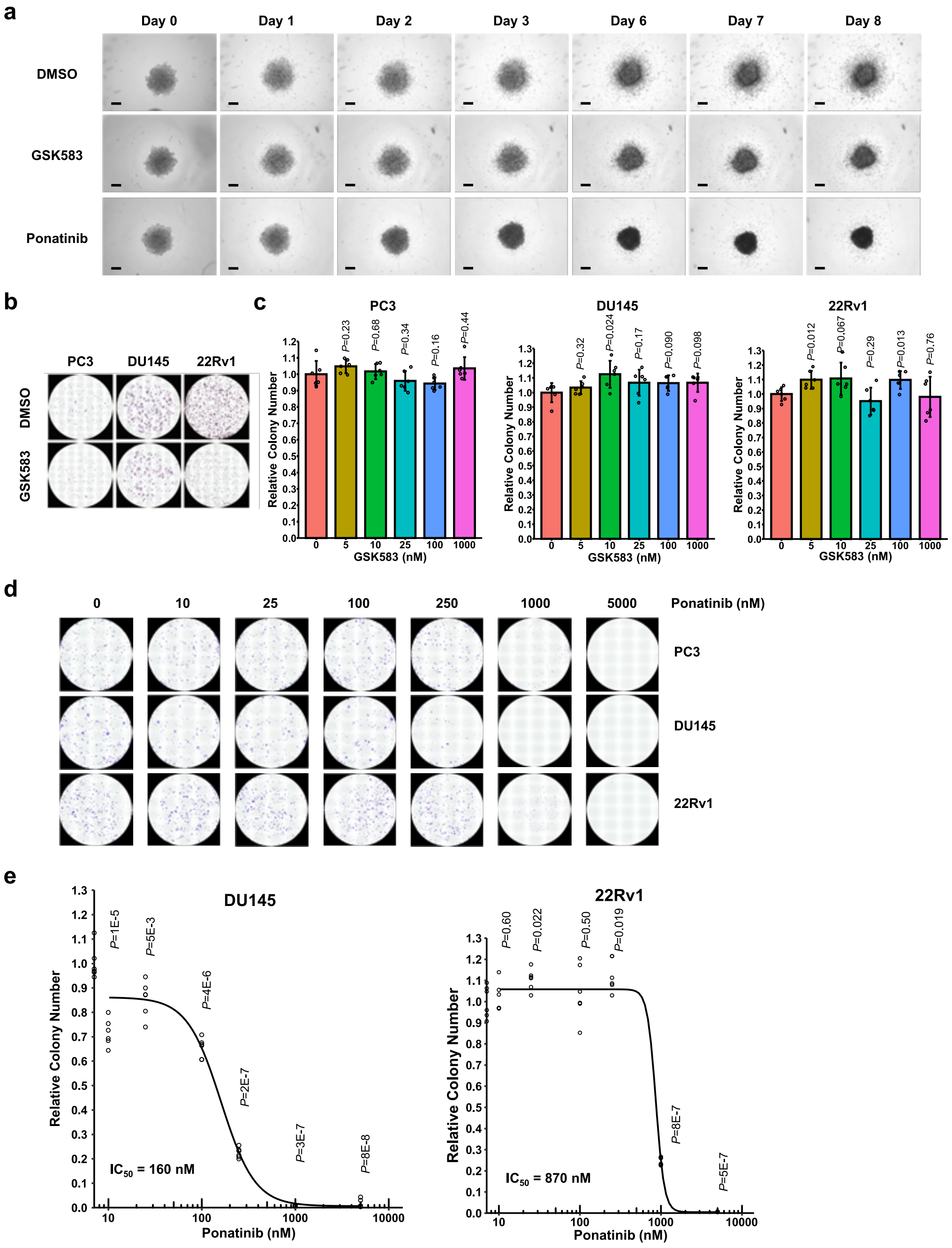

Supplemental Figure 36. Pharmacological inhibition of RIPK2 suppresses PC cell invasion and colony formation. a Representative images of 3D spheroid invasion assays of parental PC3 cells treated with vehicle control, GSK583 (10 µM), or ponatinib (5 µM). Scale bars, 300 µm. **b** Representative images of colony formation assays of parental PC cells treated with vehicle control or GSK583 (10 µM). **c** Bar plot of colony formation assays of parental PC3 (left), DU145 (middle), and 22Rv1 (right) cells treated with different concentrations of GSK583 (0-1 µM) (n = 6 biologically independent samples per group). **d** Representative images of colony formation assays of parental PC3 (top), DU145 (middle), and 22Rv1 (bottom) cells treated with different concentrations of ponatinib (0-5 µM). **e** Dose-response curves of DU145 (left) and 22Rv1 (right) cells. Cells were treated with different concentrations of ponatinib (0-5 µM) and colony formation assays were performed (n = 6 biologically independent samples per group). Nominal *p*-values were determined by unpaired two-tailed Student's *t*-test (panels c and e). Data are Mean ± SD (panel c). Experiments were repeated six times independently with similar results (panels a, b, and d).

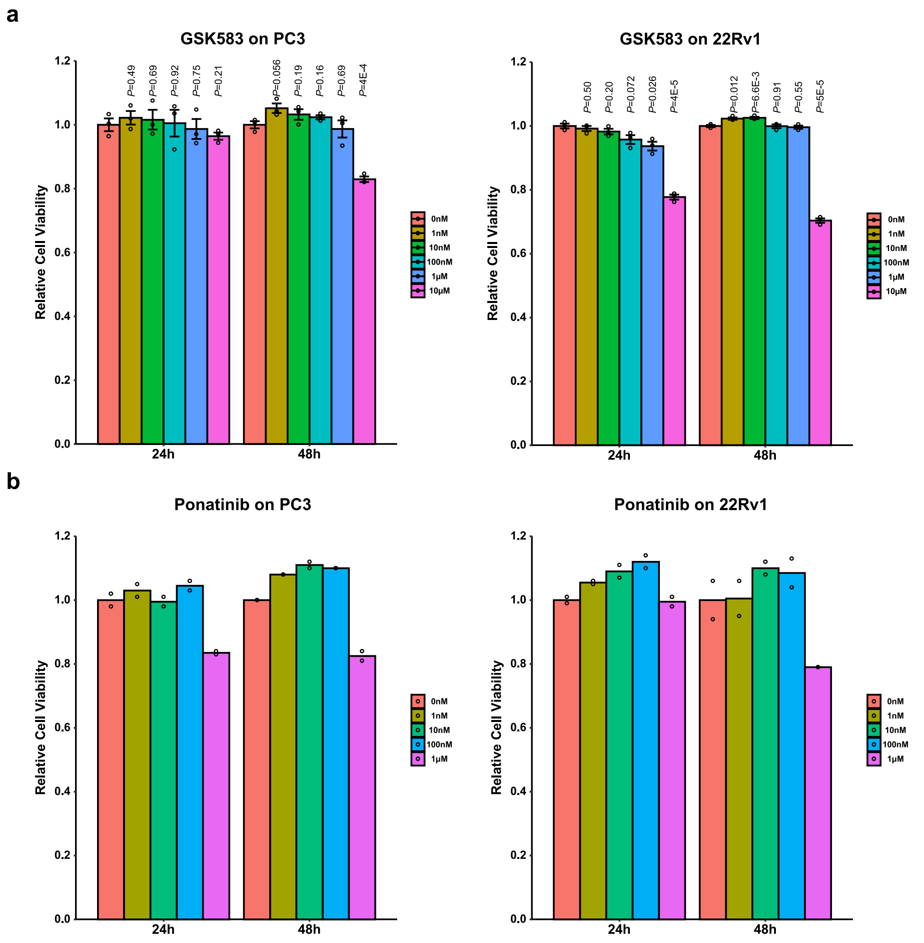

Supplementary Figure 37. GSK583 or ponatinib only has modest effects on PC cell viability when used at ≤10 or ≤1 μM, respectively. a Bar plot of relative viability of PC3 (left) and 22Rv1 (right) cells treated with the indicated concentrations of GSK583 in 10% FBS-containing cell culture medium (n = 3 biologically independent samples per group). Nominal *p*-values were determined by unpaired two-tailed Student's *t*-test. Data are Mean ± SD. **b** Bar plot of relative viability of PC3 (left) and 22Rv1 (right) cells treated with the indicated concentrations of ponatinib in 10% FBS-containing cell culture medium (n = 2 biologically independent samples per group).

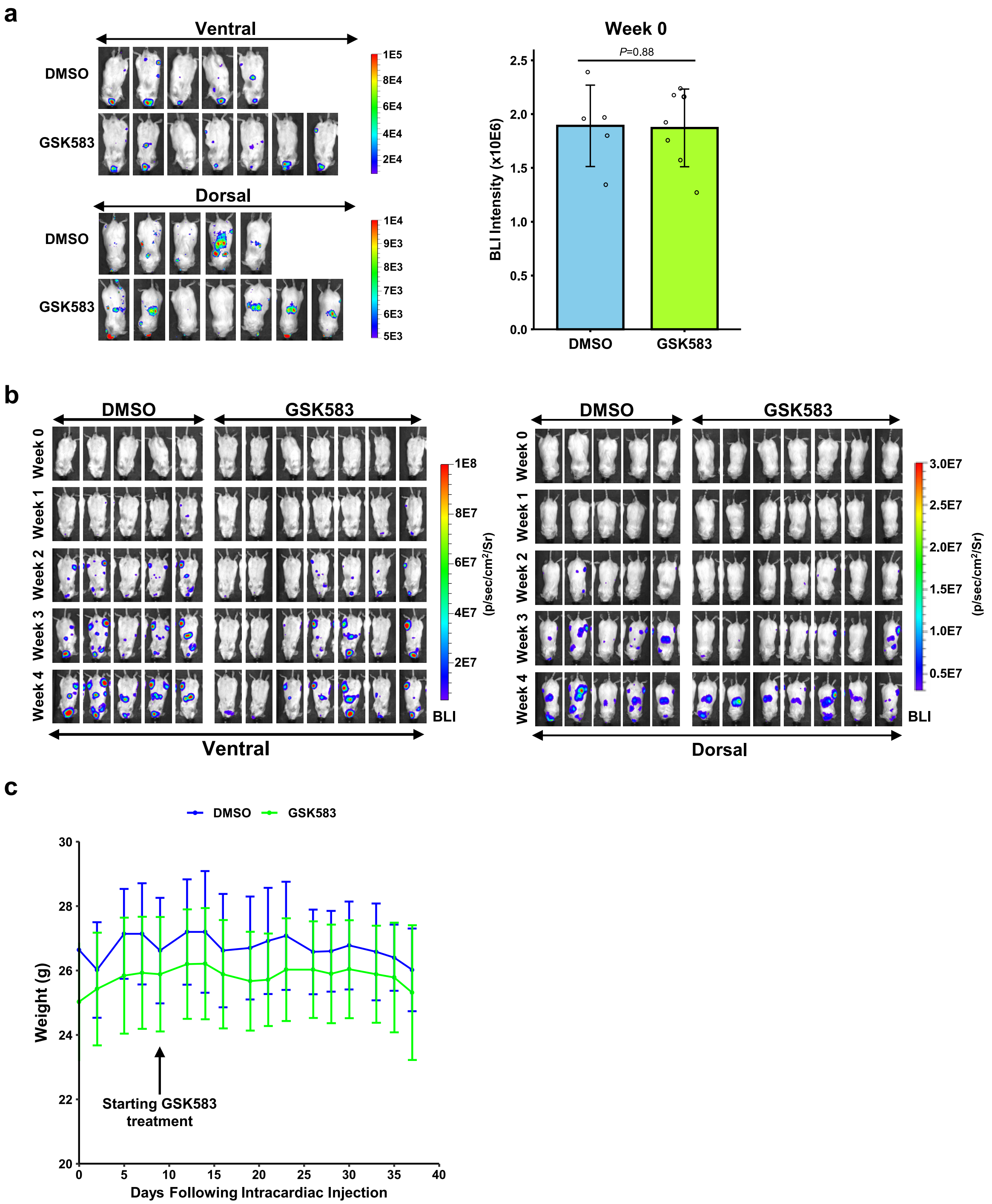

Supplementary Figure 38. GSK583 inhibits PC metastasis *in vivo*. a BLI images (left) and bar plot of the total BLI intensities (right) of mice that were randomized for GSK583 treatment on day 9 after the intracardiac injection of luciferase-tagged control 22Rv1 cells (*i.e.*, week 0 for drug treatment). Nominal *p*-value was determined by two-sided Mann-Whitney *U* test. Data are Mean ± SEM (n = 5 and 7 biologically independent mice for the control and GSK583 treatment groups, respectively). **b** BLI images of GSK583-treated mice over four weeks. **c** Changes of mouse weights following the intracardiac injection of control 22Rv1 cells (n = 5 and 7 biologically independent mice in the control and GSK583 treatment groups, respectively). Nominal *p*-values, which are all > 0.05 at different time points, were determined by two-sided Mann-Whitney *U* test. Data are Mean ± SD (n = 5 and 7 biologically independent mice in the control and GSK583 treatment groups, respectively).

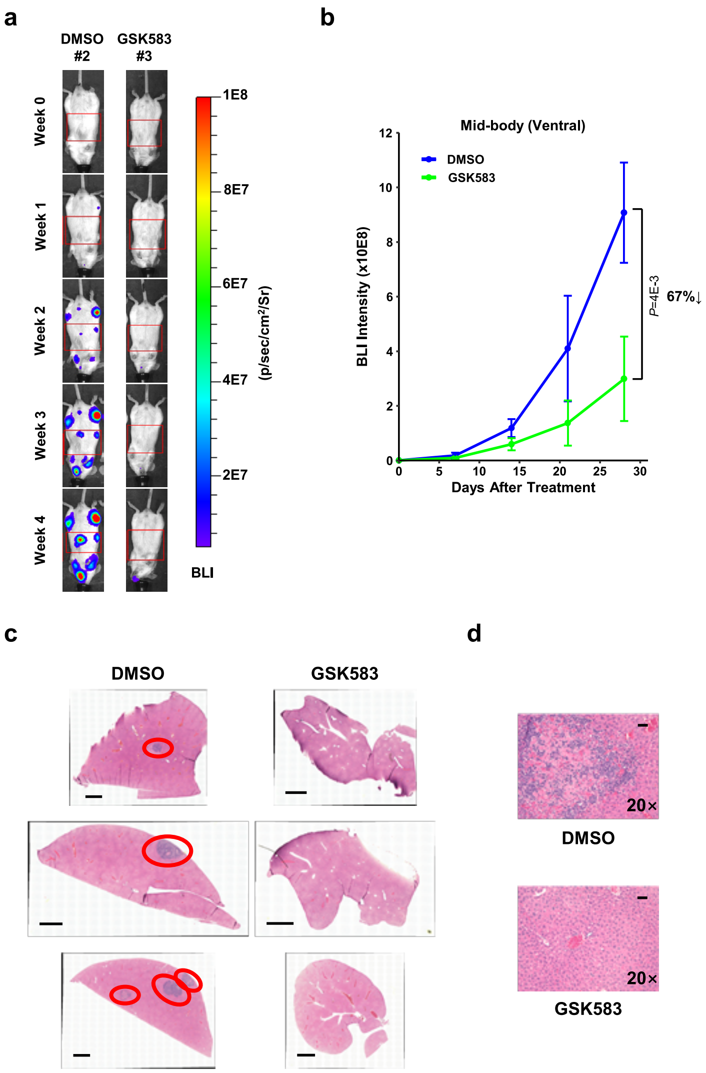

Supplementary Figure 39. GSK583 inhibits PC liver metastasis *in vivo*. a Representative BLI images of the mid-body sections (in red boxes) of mice at different weeks. Mice were intracardially injected with luciferase-tagged control 22Rv1 cells and monitored by BLI weekly. **b** Effect of GSK583 (10 mg/kg/day) on the ventral-side BLI intensities of the mid-body sections of metastasis-bearing mice. Data are Mean ± SEM (n = 5 and 7 biologically independent mice in the control and GSK583 treatment groups, respectively). **c** Representative H&E-stained images of livers from mice treated with vehicle control (left) or GSK583 (right). Scale bars, 1 mm. Liver metastases are shown in red ovals. Experiments were performed on the 12 biologically independent mice with similar results. **d** Representative H&E-stained images (20×) of livers from mice treated with vehicle control (upper) or GSK583 (lower). Scale bars, 50 μm. Experiments were repeated five times independently with similar results.

a

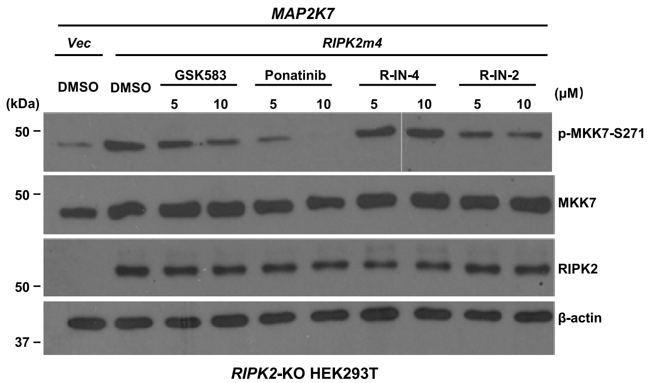

b

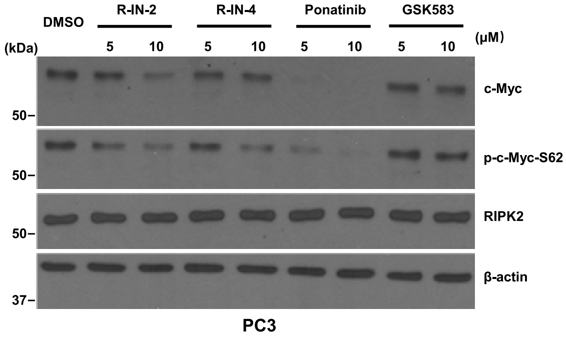

Supplementary Figure 40. Ponatinib is more potent than commercially available preclinical RIPK2 inhibitors in inactivating MKK7/c-Myc signaling. a Representative immunoblots of the indicated proteins in total lysates of *RIPK2*-KO HEK293T cells co-transfected with 0.1 µg *RIPK2m4* (or vector control) and 0.1 µg *MKK7*, which were treated with vehicle control or RIPK2 inhibitors (5 or 10 µM) in 10% FBS-containing medium for 2 h. **b** Representative immunoblots of the indicated proteins in total lysates of parental PC3 cells, which were treated with vehicle control or RIPK2 inhibitors (5 or 10 µM) in 10% FBS-containing medium for 2 h. Experiments were repeated three times independently with similar results.

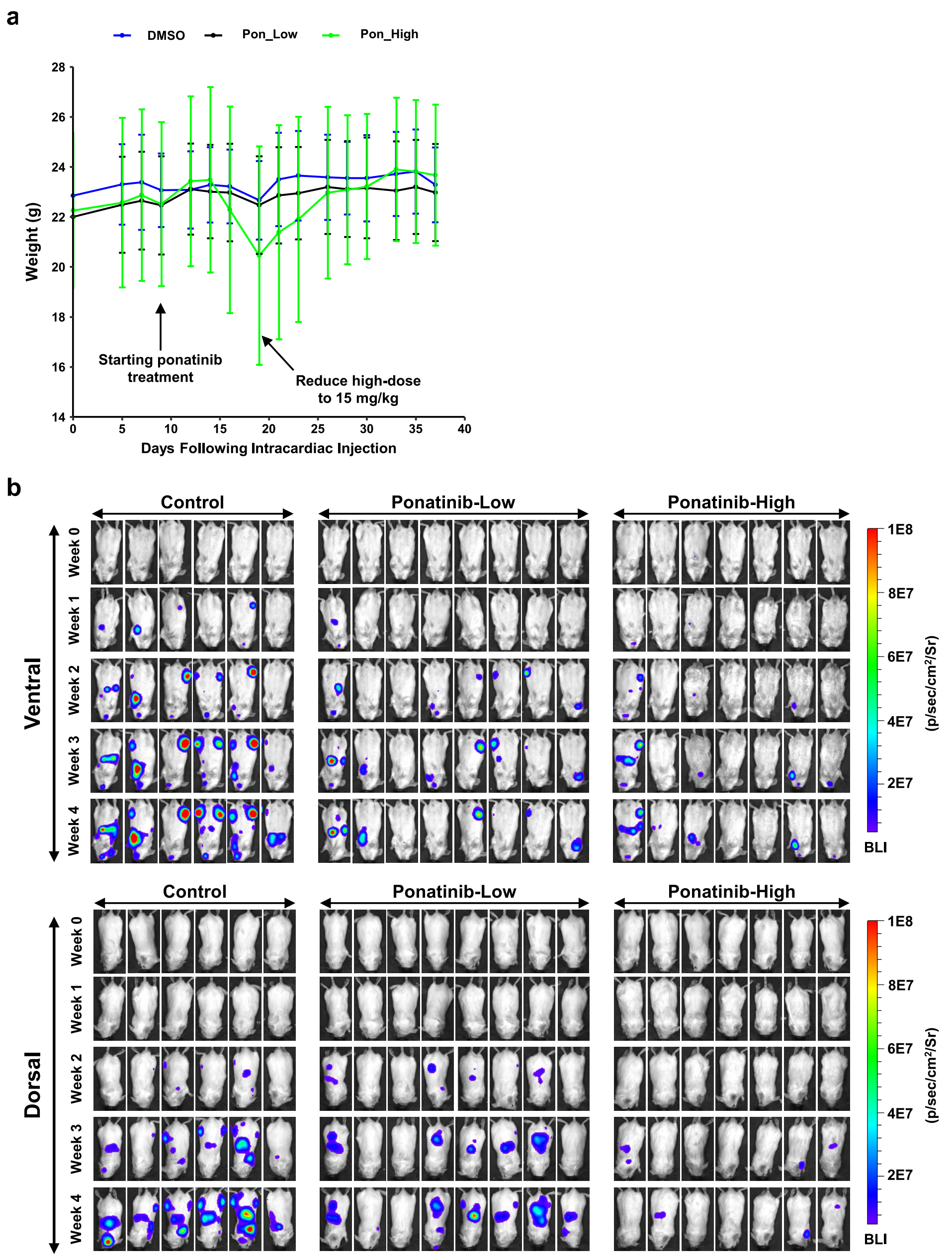

Supplementary Figure 41. Ponatinib inhibits PC metastasis in a dose-dependent fashion. **a** Line plot of mouse weights after the intracardiac injection of luciferase-tagged 22Rv1 cells (n = 6, 8, and 7 biologically independent mice in the control, ponatinib-low, and ponatinib-high groups, respectively). Nominal *p*-values, which are all > 0.05 at different time points, were determined by two-sided Mann-Whitney *U* test. Data are Mean ± SD. **b** BLI images of ponatinib-treated mice over four weeks, from the ventral (upper) and dorsal (lower) sides.

Supplementary Table 1. List of commercially available small-molecule RIPK2 inhibitors

Inhibitor Name	Type	Specificity	IC50 (nM)	Assay Method	hWB IC50 (nM)	Stage	Company	PMID	Authors	Title	Journal	Publication Year
**Ponatinib**	Active	Multi-kinase	6.7	ADP-Glo kinase assay	n/a	FDA-approved	ARIAD (Takeda)	26320862	Canning et al.	Inflammatory Signaling by NOD-RIPK2 Is Inhibited by Clinically Relevant Type II Kinase Inhibitors	Chemistry & Biology	2015
**Regorafeniib**	Active	Multi-kinase	41.0	ADP-Glo kinase assay	n/a	FDA-approved	Bayer	26320862	Canning et al.	Inflammatory Signaling by NOD-RIPK2 Is Inhibited by Clinically Relevant Type II Kinase Inhibitors	Chemistry & Biology	2015
**Sorafenib**	Active	Multi-kinase	75.4	ADP-Glo kinase assay	n/a	FDA-approved	Bayer and Onyx Pharmaceuticals	26320862	Canning et al.	Inflammatory Signaling by NOD-RIPK2 Is Inhibited by Clinically Relevant Type II Kinase Inhibitors	Chemistry & Biology	2015
**GSK583**	Active	RIPK2-selective	5.0	Fluorescence polarization assay	237	Preclinical	GlaxoSmithKline	27109867	Haile et al.	The Identification and Pharmacological Characterization of 6-(tert-Butylsulfonyl)-N-(5-fluoro-1H-indazol-3-yl)quinolin-4-amine (GSK583), a Highly Potent and Selective Inhibitor of RIP2 Kinase	Journal of Medicinal Chemistry	2016
**RIPK2-IN-4**	Active	RIPK2-selective	3.0	ADP transcreener assay	30	Preclinical	Novartis	29057049	He et al.	Identification of Potent and Selective RIPK2 Inhibitors for the Treatment of Inflammatory Diseases	ACS Medicinal Chemistry Letters	2017
**RIPK2-IN-2**	Active	RIPK2-selective	2.5	Fluorescence polarization assay	5	Preclinical	GlaxoSmithKline	30344914	Haile et al.	Identification of Quinoline-Based RIP2 Kinase Inhibitors with an Improved Therapeutic Index to the hERG Ion Channel	ACS Medicinal Chemistry Letters	2018
**GSK2983559**	Prodrug for GSK583	RIPK2-selective	5.0	Fluorescence polarization assay	26	Phase I (failed)	GlaxoSmithKline	31265286	Haile et al.	Discovery of a First-in-Class Receptor Interacting Protein 2 (RIP2) Kinase Specific Clinical Candidate, 2-((4-(Benzo[d]thiazol-5-ylamino)-6-(tert-butylsulfonyl)quinazolin-7-yl)oxy)ethyl Dihydrogen Phosphate, for the Treatment of Inflammatory Diseases	Journal of Medicinal Chemistry	2019

hWB: human whole blood cytokine release assay

Supplementary Table 2. List of primer sequences for molecular cloning

**Construct/Gene**	**5' Primer**	**3' Primer**
MKK7-6xHis	ATCCCTCGAGGGATGGCGGCGTCCTCCCTGGA	CGCGACCGGTCGCCCTGAAGAAGGGCAGGTGGG
MKK7-mCherry	ATCCCTCGAGGGATGGCGGCGTCCTCCCTGGA	CGCGGGATCCGGCCTGAAGAAGGGCAGGTGGG
NES-RIPK2m4-3xFLAG	ATCCCTCGAGGCCACCATGCTACCGCCGCTGGAAAGACTGACTCTGGGAGGCGGGATGAACGGGGAGGCCATC	CGCGACCGGTCCTTACTTATCGTCATCGTCTTTGTAATCAATATCATGATCCTTGTAGTCTCCGTCGTGGTCCTTATAGTCCATGCTTTTATTTTGAAGTAA
NLS-RIPK2m4-3xFLAG	ATCCCTCGAGGCCACCATGCCAAAAAAGAAAAGAAAAGTTATGAACGGGGAGGCCATC	CGCGACCGGTCCTTACTTATCGTCATCGTCTTTGTAATCAATATCATGATCCTTGTAGTCTCCGTCGTGGTCCTTATAGTCCATGCTTTTATTTTGAAGTAA
RIPK2m4-3xFLAG	ATCCCTCGAGGGATGAACGGGGAGGCCATCT	CGCGACCGGTCCTTACTTATCGTCATCGTCTTTGTAATCAATATCATGATCCTTGTAGTCTCCGTCGTGGTCCTTATAGTCCATGCTTTTATTTTGAAGTAA
RIPK2m4-delta CARD Domain-3xFLAG	ATCCCTCGAGGGATGAACGGGGAGGCCATCT	CGCGACCGGTCCTTACTTATCGTCATCGTCTTTGTAATCAATATCATGATCCTTGTAGTCTCCGTCGTGGTCCTTATAGTCCAGACGTTCTGAGTTTCCTGC
RIPK2m4-delta Kinase Domain-3xFLAG	ATCCCTCGAGGGATGAGAACATTTGAAGAGATAACT	CGCGACCGGTCCTTACTTATC
RIPK2m4-eGFP	ATCCGCTAGCGCCACCATGAACGGGGAGGCCATCT	CGCGACCGGTCCCATGCTTTTATTTTGAAGTAAAT
RIPK2m4	GGCTGTTTTCTGGATAACACTTCCCAGGTTATTACAGCATAGCTATATATATCGTGCTTGAT	ATCAAGCACGATATATATAGCTATGCTGTAATAACCTGGGAAGTGTTATCCAGAAAACAGCC
MYC	GAACAAGAAGATGAGGAAGAA	CAGAAGGTGATCCAGACT
GAPDH	GGTGGTCTCCTCTGACTTCAACA	GTTGCTGTAGCCAAATTCGTTGT

Supplementary Table 3. List of sgRNA sequences for CRISPR/Cas9 knockout

Targets	gRNA #	Sequence	Domain	Source	sgRNA target region in CDS	Region and Domain in Protein	Exon # in gene
RIPK2	10	ACAGCTATGCAGTTATCACA	Kinase Domain	Addgene	640-657	52-882 (Kinase)	3
RIPK2	2	AGTAGCAAATCTGCACCAGA	Kinase Domain	Self	538-557	52-882 (Kinase)	2
RIPK2	3	GCTACTTCGTGACTGTGAGA	Kinase Domain	Self	524-543	52-882 (Kinase)	2
MKK7	1	ACGGGCTACCTGACCATCGG	beta strand before kinase domain	Self	307-326	310-324 (Beta-strand)	2
MKK7	2	CAAAGCACTGCACGATGTAG	Kinase Domain	Self	531-550	358-1140 (Kinase)	5
PRKDC	1	GTTCTCAGAAACGATCAACA	Between HEAT2 Domain and TPR1 Domain	Self	4686-4705	Between HEAT2 (3010- 3120) and TPR1 (5167-5628) domains	36
PRKDC	2	GCACATCATCATGCACCGTG	Between TPR1 Domain and FAT Domain	Self	6222-6241	Between TPR1 (5167-5628) and FAT (8716-10617) domains	47
Control gRNA	1	GAACTCAACCAGAGGGCCAA		Addgene			
