# Supplementary material for: Receptor-interacting protein kinase 2 (RIPK2) stabilizes c-Myc and is a therapeutic target in prostate cancer metastasis
Source: Nat Commun. 2022 Feb 3;13:669. doi: 10.1038/s41467-022-28340-6 (PMC8813925; doi:10.1038/s41467-022-28340-6)
Supplement: Supplementary file 3 — Description of Additional Supplementary Files [file 41467_2022_28340_MOESM3_ESM.pdf]

Description of Additional Supplementary Files

Supplementary Data 1. Labelfree proteomic identification and quantification of proteins in control and RIPK2-KO PC3 cells.

Supplementary Data 2. List of the 652 protein groups that are differentially expressed in control and RIPK2-KO PC3 cells.

Supplementary Data 3. List of the 219 protein candidates that associate with the kinase domain and no other regions of RIPK2.

Supplementary Data 4. Labelfree phosphoproteomic comparison of control and RIPK2-KO PC3 cells.

Supplementary Data 5. List of kinases whose inferred kinase activities are regulated by RIPK2 in PC3 cells.
