# Supplementary material for: Receptor-interacting protein kinase 2 (RIPK2) stabilizes c-Myc and is a therapeutic target in prostate cancer metastasis
Source: Nat Commun. 2022 Feb 3;13:669. doi: 10.1038/s41467-022-28340-6 (PMC8813925; doi:10.1038/s41467-022-28340-6)
Supplement: Supplementary file 9 — Reporting summary [file 41467_2022_28340_MOESM9_ESM.pdf]

natureresearch

Corresponding author(s): Wei Yang

Last updated by author(s): Dec 22, 2021

Reporting Summary

Nature Research wishes to improve the reproducibility of the work that we publish. This form provides structure for consistency and transparency in reporting. For further information on Nature Research policies, see Authors & Referees and the Editorial Policy Checklist .

Statistics

For all statistical analyses, confirm that the following items are present in the figure legend, table legend, main text, or Methods section.

n/a	Confirmed	
☐	☒	The exact sample size (*n*) for each experimental group/condition, given as a discrete number and unit of measurement
☐	☒	A statement on whether measurements were taken from distinct samples or whether the same sample was measured repeatedly
☐	☒	The statistical test(s) used AND whether they are one- or two-sided<br>*Only common tests should be described solely by name; describe more complex techniques in the Methods section.*
☒	☐	A description of all covariates tested
☐	☒	A description of any assumptions or corrections, such as tests of normality and adjustment for multiple comparisons
☐	☒	A full description of the statistical parameters including central tendency (e.g. means) or other basic estimates (e.g. regression coefficient) AND variation (e.g. standard deviation) or associated estimates of uncertainty (e.g. confidence intervals)
☐	☒	For null hypothesis testing, the test statistic (e.g. *F*, *t*, *r*) with confidence intervals, effect sizes, degrees of freedom and *P* value noted<br>*Give P values as exact values whenever suitable.*
☒	☐	For Bayesian analysis, information on the choice of priors and Markov chain Monte Carlo settings
☒	☐	For hierarchical and complex designs, identification of the appropriate level for tests and full reporting of outcomes
☐	☒	Estimates of effect sizes (e.g. Cohen's *d*, Pearson's *r*), indicating how they were calculated

Our web collection on statistics for biologists contains articles on many of the points above.

Software and code

Policy information about availability of computer code

Data collection	Xcalibur (v4.1) (Thermo Scientific)
Data analysis	BD FACDiva (BD Biosciences, v8.0.1), Living Image software (Caliper Life Sciences, v4.3.1), MaxQuant (v1.5.5.1) , Perseus (v1.6.6.0), Leica Tissue IA software package (Leica Biosystems, v1.0), Image J (v1.52p), Rosetta(v3.11), ZDOCK (2.3.2), R (v3.6.2).

For manuscripts utilizing custom algorithms or software that are central to the research but not yet described in published literature, software must be made available to editors/reviewers. We strongly encourage code deposition in a community repository (e.g. GitHub). See the Nature Research guidelines for submitting code & software for further information.

Data

Policy information about availability of data

All manuscripts must include a data availability statement. This statement should provide the following information, where applicable:

- Accession codes, unique identifiers, or web links for publicly available datasets
- A list of figures that have associated raw data
- A description of any restrictions on data availability

The described label-free proteomics, interactome, phosphoproteomics data have been deposited in the PRIDE repository under the following accession numbers: PXD018890 (https://www.ebi.ac.uk/pride/archive/projects/PXD018890) (proteomics), PXD018870 (https://www.ebi.ac.uk/pride/archive/projects/PXD018870) (interactome), PXD018871 (https://www.ebi.ac.uk/pride/archive/projects/PXD018871) (phosphoproteomics). The cBioPortal data are available for download from cbioportal.org. The PCTA data are available from thepcta.org. The Pharos data are available from pharos.nih.gov. The MSigDB (v7.4) data are available from https://www.gsea-msigdb.org/gsea/msigdb/. The CCLE data are available from depmap.org. The Uniprot_Human data are available from uniprot.org. Source data are provided with this paper.

Field-specific reporting

Please select the one below that is the best fit for your research. If you are not sure, read the appropriate sections before making your selection.

☒ Life sciences ☐ Behavioural & social sciences ☐ Ecological, evolutionary & environmental sciences

For a reference copy of the document with all sections, see nature.com/documents/nr-reporting-summary-flat.pdf

Life sciences study design

All studies must disclose on these points even when the disclosure is negative.

Sample size	The sample size for each experiment is specified in the Methods section and figure legends. For animal studies, sample sizes were determined based on similar published studies (Chu et al., Endocrine-Related Cancer, 2014, 21(2): 311-26; Li et al., Oncotarget, 2016, 7(11): 12869-84). For ethical reasons, minimum numbers of animals required to achieve the scientific goals were used. At least 5 animals per group were used to allow for basic statistical inference.
Data exclusions	Outlier RIPK2-interactome samples were determined by unsupervised clustering of log2-transformed label-free quantification (LFQ) intensities, followed by visual inspection.
Replication	For each experiment, the number of biological replicates is reported in the figure or figure legend.
Randomization	All cell samples and animals were randomly allocated to experimental and control groups. All animal experiments use mice with matched age.
Blinding	All experiments were assigned into groups including relevant controls. Data analysis was performed by investigators blinded to the experimental conditions.

Reporting for specific materials, systems and methods

We require information from authors about some types of materials, experimental systems and methods used in many studies. Here, indicate whether each material, system or method listed is relevant to your study. If you are not sure if a list item applies to your research, read the appropriate section before selecting a response.

Materials & experimental systems

n/a	Involved in the study	
☐	☒	Antibodies
☐	☒	Eukaryotic cell lines
☒	☐	Palaeontology
☐	☒	Animals and other organisms
☒	☐	Human research participants
☒	☐	Clinical data

Methods

n/a	Involved in the study	
☒	☐	ChIP-seq
☐	☒	Flow cytometry
☒	☐	MRI-based neuroimaging

Antibodies

Antibodies used

For western blotting, the following antibodies were used: RIPK2 (1:1,000, Cell Signaling Technology #4142 or Santa Cruz Biotechnology #sc-166765), c-Myc (1:5,000, Abcam #ab32072), phospho-c-Myc (Ser62) (1:1,000, Cell Signaling Technology #13748S), ubiquitin (K48-linkage specific) (1:1,000, Cell Signaling Technology, #12805), FLAG (1:5,000, Sigma #F1804), MKK7 (1:1,000, Cell Signaling Technology #4172S or 1:2,000, Santa Cruz Biotechnology #sc-25288), MKK7 (phospho-Ser271) (1:1,000, Aviva Systems Biology #OAAF05547), JNK (1:1,000, Cell Signaling Technology #9252S or 1:2,000 Santa Cruz Biotechnology sc-7345), JNK (phospho-T183/Y185) (1:1,000, Cell Signaling Technology #9251S), mCherry (1:1,000, Abcam #ab213511), GFP (1:1,000, Abcam #ab290), β-actin (1:5,000, Sigma Aldrich #A5441), and GAPDH (1:1,000, Cell Signaling Technology #3683). Signal was visualized with anti-mouse or anti-rabbit IgG, secondary HRP-conjugated antibodies (1:5,000, Cell Signaling Technology #7074S or #7076S) and chemiluminescent detection.

For immunoprecipitation, the following antibodies (3 ug) were used: anti-FLAG antibody (Sigma Aldrich, #F1804), IgG (Millipore, #12-371), and anti-c-Myc antibody (Abcam #ab32072).

For immunohistochemical staining, anti-RIPK2 antibody (Sigma Aldrich #HPA015273) was used at 1:100 dilution.

For immunofluorescence imaging, the following antibodies were used: anti-FLAG primary antibody (1:500, Sigma Aldrich #F1804) and fluorochrome-conjugated secondary antibody, i.e., anti-mouse IgG (H+L), F(ab')2 Fragment (Alexa Fluor 488 Conjugate) (1:1,000, Cell Signaling Technology #4408).

For proximity ligation assay, the following antibodies were used: rabbit anti-RIPK2 (1:500, Cell Signaling Technology, #4142),

mouse anti-MKK7 (1:100, Santa Cruz Biotechnology, #sc-25288), and mouse anti-PRKDC (1:100, Santa Cruz Biotechnology, #sc-5282).

Validation

Antibodies were only used for the applications and organisms as indicated by the manufacturers (see below). All the antibodies have been cited >10 times.

RIP2 (D10B11) Rabbit mAb #4142: https://www.cellsignal.com/products/primary-antibodies/rip2-d10b11-rabbit-mab/4142. The antibody has been validated for western blotting in publications (PMID: 32954645; 31350258). The antibody has also been validated in this study by comparing RIPK2-knockout (KO) and RIPK2-overexpression (OE) with controls (Figs. 2a and 2f). For PLA assays, appropriate controls are included (Fig. 6a).

Phospho-c-Myc (Ser62) (E1J4K) Rabbit mAb #13748S: https://www.cellsignal.com/products/primary-antibodies/phospho-c-myc-ser62-e1j4k-rabbit-mab/13748?site-search-type=Products&N=4294956287&Ntt=13748s&fromPage=plp&_requestid=473055. The antibody has been validated for western blotting in publications (PMID: 33298911; 32049046).

K48-linkage Specific Polyubiquitin (D9D5) Rabbit mAb (HRP Conjugate) #12805: https://www.cellsignal.com/products/antibody-conjugates/k48-linkage-specific-polyubiquitin-d9d5-rabbit-mab-hrp-conjugate/12805. The antibody has been validated for western blotting in publications (PMID: 33771975; 30429547).

MKK7 Antibody #4172: https://www.cellsignal.com/products/primary-antibodies/mkk7-antibody/4172?site-search-type=Products&N=4294956287&Ntt=4172s&fromPage=plp&_requestid=473298. The antibody has been validated for western blotting in publications (PMID: 34502457; 26270349).

SAPK/JNK Antibody #9252: https://www.cellsignal.com/products/primary-antibodies/sapk-jnk-antibody/9252?site-search-type=Products&N=4294956287&Ntt=9252s&fromPage=plp&_requestid=473351. The antibody has been validated for western blotting in publications (PMID: 31953436; 30610188).

Phospho-SAPK/JNK (Thr183/Tyr185) Antibody #9251: https://www.cellsignal.com/products/primary-antibodies/phospho-sapk-jnk-thr183-tyr185-antibody/9251?site-search-type=Products&N=4294956287&Ntt=9251s&fromPage=plp&_requestid=473393. The antibody has been validated for western blotting in publications (PMID: 31953436; 30610188).

GAPDH (14C10) Rabbit mAb (HRP Conjugate) #3683: https://www.cellsignal.com/products/antibody-conjugates/gapdh-14c10-rabbit-mab-hrp-conjugate/3683. The antibody has been validated for western blotting in publications (PMID: 29808028; 29057869).

Anti-rabbit IgG, HRP-linked Antibody #7074: https://www.cellsignal.com/products/secondary-antibodies/anti-rabbit-igg-hrp-linked-antibody/7074?site-search-type=Products&N=4294956287&Ntt=7074s&fromPage=plp&_requestid=473487. The antibody has been validated for western blotting in publications (PMID: 32958754; 32843625).

Anti-mouse IgG, HRP-linked Antibody #7076: https://www.cellsignal.com/products/secondary-antibodies/anti-mouse-igg-hrp-linked-antibody/7076?site-search-type=Products&N=4294956287&Ntt=7076s&fromPage=plp&_requestid=473512. The antibody has been validated for western blotting in publications (PMID: 32843618; 31974375).

Anti-mouse IgG (H+L), F(ab')2 Fragment (Alexa Fluor 488 Conjugate) #4408: https://www.cellsignal.com/products/secondary-antibodies/anti-mouse-igg-h-l-f-ab-2-fragment-alexa-fluor-488-conjugate/4408. The antibody has been validated for immunofluorescence in publications (PMID: 32958754; 31959826).

Anti-RICK (RIPK2) Antibody (A-10) sc-166765: https://www.scbt.com/p/rick-antibody-a-10?requestFrom=search. Also validated in this study by comparing RIPK2-KO and RIPK2-OE with controls. The antibody has been validated for western blotting in publications (PMID: 31350258; 34152391).

Anti-MEK-7 Antibody (E-7) sc-25288: https://www.scbt.com/p/mek-7-antibody-e-7?requestFrom=search. The antibody has been validated for western blotting in publications (PMID: 28111074; 31331998). It has also been validated in this study by comparing MKK7-KO with control (Fig. 6i and Supplementary Fig. 30a). For PLA assays, appropriate controls are included (Figs. 6a and 7c).

Anti-JNK Antibody (D-2) sc-7345: https://www.scbt.com/p/jnk-antibody-d-2?requestFrom=search. The antibody has been validated for western blotting in publications (PMID: 32493999; 29057873).

Anti-DNA-PKcs Antibody (G-4) sc-5282: https://www.scbt.com/p/dna-pkcs-antibody-g-4?requestFrom=search. The antibody has been validated for western blotting in publications (PMID: 31444354; 31086186). The antibody has also been validated in this study by comparing PRKDC-KO with control (Fig. 6i). For PLA assays, appropriate controls are included (Supplementary Fig. 30c).

Recombinant Anti-c-Myc antibody [Y69] (ab32072): https://www.abcam.com/c-myc-antibody-y69-ab32072.html. The antibody has been validated for western blotting in publications (PMID: 32814769; 31953400). The antibody has been validated for immunoprecipitation in publications (PMID: 33038488; 33748098).

Recombinant Anti-mCherry antibody [EPR20579] (ab213511): https://www.abcam.com/mcherry-antibody-epr20579-ab213511.html. The antibody has been validated for western blotting in publications (PMID: 34172933; 33476576).

Anti-GFP antibody (ab290): https://www.abcam.com/gfp-antibody-ab290.html. The antibody has been validated for western

blotting in publications (PMID: 33594072; 33602938).

Monoclonal anti-FLAG M2 antibody produced in mouse (F1804): https://www.sigmaaldrich.com/US/en/product/sigma/f1804. The antibody has been validated for western blotting (PMID: 34862394; 34893641), for immunoprecipitation (PMID: 32312989; 33247121), and for immunofluorescence (PMID: 29335436; 29192139).

Monoclonal anti-beta-actin antibody produced in mouse (A5441): https://www.sigmaaldrich.com/US/en/search/beta-actin-antibody?focus=products&page=1&perPage=30&sort=relevance&term=beta-actin%20antibody&type=product. The antibody has been validated for western blotting in publications (PMID: 34782646; 34584081).

Anti-RIPK2 antibody produced in rabbit HPA015273: https://www.sigmaaldrich.com/US/en/search/hpa015273?focus=products&page=1&perPage=30&sort=relevance&term=HPA015273&type=product. The antibody has been validated for IHC in this study by comparing RIPK2-KO with control (supplementary Fig. 1g).

Normal Mouse IgG 12-371: https://www.emdmillipore.com/US/en/product/Normal-Mouse-IgG,MM_NF-12-371. The IgG has been validated for immunoprecipitation in publications (PMID: 34446700; 32807793).

Eukaryotic cell lines

Policy information about cell lines

Cell line source(s)	PC3, DU145, 22Rv1, LNCaP, RWPE-1, RWPE-2, HEK293T, and HeLa cells were obtained from the American Type Culture Collection (ATCC). RIPK2-KO, MKK7-KO, PRKDC-KO, RIPK2/MKK7-DKO, RIPK2/PRKDC-DKO cell lines were generated in the lab with the CRISPR/Cas9 methodology.
Authentication	All the cell lines were authenticated using the Promega PowerPlex 16 system DNA typing (Laragen).
Mycoplasma contamination	Mycoplasma contamination was routinely monitored using the MycoAlert PLUS Mycoplasma Detection Kit (Lonza, #LT07-118) for all the used cell lines.
Commonly misidentified lines (See ICLAC register)	No commonly misidentified cell lines were used.

Animals and other organisms

Policy information about studies involving animals; ARRIVE guidelines recommended for reporting animal research

Laboratory animals	Male SCID/Beige mice (Charles River; strain code 250; CB17.Cg-PrkdcscidLystbg-J/Crl) between 7-8-weeks at beginning of study (study duration 4-5 weeks). Mice were housed at 74°F (±2°F) with ambient humidity. The light cycle of animal rooms was 10 h of light and 14 h of dark.
Wild animals	Study did not involve wild animals.
Field-collected samples	Study did not involve field collected samples.
Ethics oversight	All experimental protocols and procedures were approved by the Institutional Animal Care and Use Committee (IACUC) at Cedars-Sinai Medical Center and the Animal Care and Use Review Office at the Department of Defense. All relevant ethical regulations, standards, and norms were rigorously adhered to.

Note that full information on the approval of the study protocol must also be provided in the manuscript.

Flow Cytometry

Plots

Confirm that:

- [x] The axis labels state the marker and fluorochrome used (e.g. CD4-FITC).
- [x] The axis scales are clearly visible. Include numbers along axes only for bottom left plot of group (a 'group' is an analysis of identical markers).
- [x] All plots are contour plots with outliers or pseudocolor plots.
- [x] A numerical value for number of cells or percentage (with statistics) is provided.

Methodology

Sample preparation	CMV-copGFP-Luc2-Puro, psPAX2 and pMD2.G plasmids were co-transfected into HEK293T cells to produce lentivirus containing copGFP-labelled firefly luciferase. Control and RIPK2-knockout (RIPK2-KO) 22Rv1 cells were infected with lentivirus containing copGFP-labelled luciferase supplemented with 10 µg/mL polybrene. Two weeks later, cells were trypsinized, washed with DPBS for one time and resuspended in DPBS. Cells were filtered by 35 µm cell strainer before sorting the GFP-positive control and RIPK2-KO 22Rv1 cells using same FITC-A gate. Parental 22Rv1 cells without GFP were used as negative control.

Instrument	FACSAria III (BD Biosciences)
Software	BD FACSDiva (v8.0.1)
Cell population abundance	Parental 22Rv1 cells without GFP were used as negative control and the proportion of GFP positive cells is 0%. The proportion of GFP positive cells in luciferase stably transfected control 22Rv1 cells was 67.2% and in RIPK2-KO 22Rv1 cells was 84.3%. 0.9 million cells were sorted for both GFP positive control and RIPK2-KO 22Rv1 cells.
Gating strategy	Cells were defined based on FSC-A and SSC-A profile. Singlets were gated based on the pattern of SSC-H vs SSC-W and then FSC-H vs FSC-W. GFP positive population was sub-gated based on FITC-A vs SSC-A using parental 22Rv1 cells without GFP to define the negative population.

☒ Tick this box to confirm that a figure exemplifying the gating strategy is provided in the Supplementary Information.
